# Direct Measurement of the Diffusion Coefficient of Adhesives from Moisture Distribution in Adhesive Layers Using Near-Infrared Spectroscopy

**DOI:** 10.1021/acsami.4c11286

**Published:** 2024-09-28

**Authors:** Jin-Woo Han, Yu Sekiguchi, Kazumasa Shimamoto, Haruhisa Akiyama, Chiaki Sato

**Affiliations:** † Department of Mechanical Engineering, Tokyo Institute of Technology, 4259 Nagatsuta-cho, Midori-ku, Yokohama, Kanagawa 226-8501, Japan; ‡ Institute of Innovative Research, Tokyo Institute of Technology, 4259 Nagatsuta-cho, Midori-ku, Yokohama, Kanagawa 226-8501, Japan; § Nanomaterials Research Institute, National Institute of Advanced Industrial Science and Technology (AIST), 1-1-1 Higashi, Tsukuba, Ibaraki 305-8565, Japan

**Keywords:** epoxy adhesive, moisture absorption, nondestructive testing, near-infrared analysis, water resistance

## Abstract

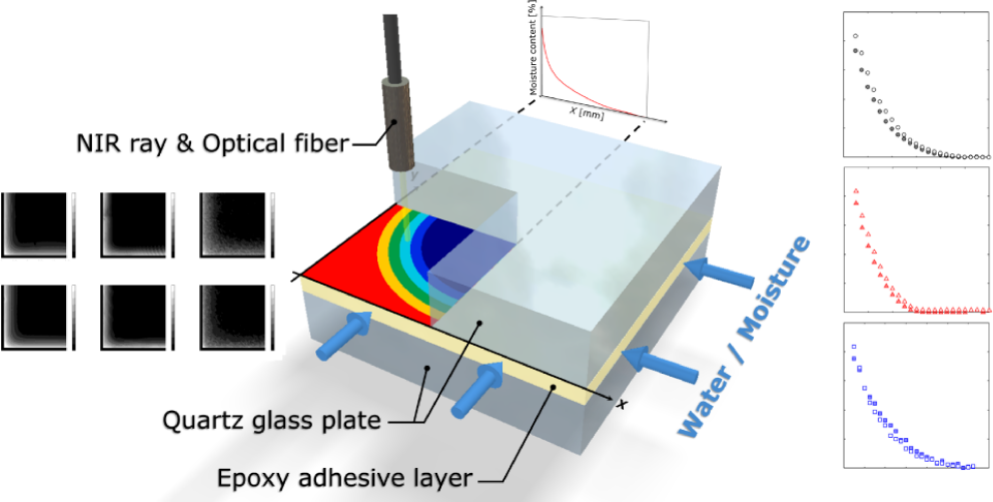

In this study, we used near-infrared spectroscopy to measure the moisture penetration in epoxy adhesives and investigated the difference in the diffusion coefficients between the bulk and the adhesive layer. Moisture diffusion was evaluated under 100% RH and water immersion conditions. First, the effects of the curing agents and additives on moisture diffusion in the bulk were gravimetrically evaluated using epoxy-coated quartz glass plates. Different diffusion behaviors were observed depending on the curing agent used. The presence of additives resulted in higher diffusion coefficients, whereas the overall moisture content was low. Next, the moisture distribution in the adhesive layer was visualized using a specimen sandwiched between the quartz glass plates, and the diffusion coefficient of the adhesive layer was calculated. The diffusion coefficient in the adhesive layer was larger than that in the bulk. For adhesives cured with hydrophobic diamine, the diffusion coefficient within the adhesive layer increased by approximately 1.5 times compared with that in the bulk, regardless of the exposure environment. The adhesive, composed of a resin, Dicyandiamide, and additives, showed a 2-fold increase in the diffusion coefficient under high-humidity exposure conditions but no significant change under the water immersion condition. Therefore, these results suggest that, for an accurate analysis of moisture distribution, it is important to measure the diffusion coefficient of the adhesive layer directly rather than using the diffusion coefficient of the material itself.

## 1 Introduction

Adhesive bonding technology has been used in manufacturing owing to its advantages such as uniform load transfer, lightweight, and capability to bond dissimilar materials.^1,2^ Epoxy adhesives are widely used as structural adhesives in the aerospace and automotive industries owing to their excellent mechanical properties and high chemical and thermal resistance.^3−6^ However, adhesive joints used in these applications are frequently exposed to high humidity or water environments.^7−10^

Moisture has a significant long-term effect on the performance of adhesive joints. When moisture is absorbed into the polymer matrix of adhesives, the interaction between the polymer chains weakens, leading to decreased strength.^11^ In particular, in aqueous environments, the leaching of uncured resin and additives from the adhesive can accelerate strength reduction.^12,13^ The adhesive layer, sandwiched between the substrates, allows moisture to diffuse from the edges, resulting in moisture distribution, inducing stress derived from the swelling or deformation of the adhesive, and potentially resulting in adhesive failure.^14^ Moreover, moisture tends to accumulate near the interface between the substrate and the adhesive layer, reducing the interfacial strength.^15,16^ To predict mechanical degradation and failure, a combination of several effects must be considered, including softening due to moisture absorption, strength degradation due to hydrolysis, and a decrease in interfacial strength due to corrosion. However, as a first step toward understanding the effect of moisture on the adhesive layer, accurate measurement of the moisture distribution in adhesive joints is crucial and determination of the diffusion coefficient is absolutely necessary. Therefore, we focused only on the water absorption properties of the materials.

Moisture diffusion in the adhesive layer is generally predicted using the diffusion coefficient obtained from the gravimetric changes in the bulk adhesive, assuming simple Fickian diffusion.^17,18^ However, some studies have indicated that diffusion may be faster near the interface than at the center of the adhesive layer,^16,19^ potentially leading to faster overall moisture diffusion than that in the bulk. The diffusion behavior of some adhesives deviates from simple Fickian diffusion, exhibiting non-Fickian or anomalous diffusion, owing to factors such as polymer relaxation, swelling, and hydrogen bonding.^14,20^ Therefore, relying only on simple Fickian diffusion for predicting moisture distribution has certain limitations. To address this issue, models that analyze dual-stage diffusion by combining the moisture concentration distributions obtained from two separate Fickian diffusion analyses have been proposed.^21^ However, numerical models require experimental validation of the moisture distribution within the adhesive layer.

The adhesive layer is sandwiched between the substrates and cannot be observed directly. Furthermore, the adhesive layer generally has a thickness of several hundred micrometers, and the amount of moisture that permeates the adhesive layer is small. Therefore, experimental observation of moisture penetration into the adhesive layer requires a nondestructive, high-resolution measurement method. Experimental approaches include measuring swelling-induced deformation,^22,23^ monitoring modulus changes using electrochemical impedance spectroscopy,^24^ using Fourier-transform infrared transmission spectroscopy to measure D_2_O diffusion in adhesive layers,^25,26^ and using near-infrared spectroscopy (NIRS).^17^ However, due to the limitation in the measurement methods, only a few experimental studies visualized moisture distribution and observed its diffusion process in adhesive layers. Therefore, moisture penetration in the adhesive layer is still mysterious and phenomena that need to be elucidated are ubiquitous.

In this study, fiber-type NIRS and an automatic *XY*-axis stage were used to determine the moisture distribution within the adhesive layer. Three types of adhesives were used to investigate the influence of curing agents and additives on moisture diffusion, and two environmental conditions—(A) exposure to 100% relative humidity (RH) at room temperature and (B) immersion in purified water at room temperature—were introduced. First, moisture absorption in the epoxy adhesives was investigated using adhesive-coated glass plates (open-face specimens). Gravimetric and NIRS spectral changes were compared. Next, the moisture distribution within the adhesive layer sandwiched between the quartz glass plates (closed specimen) was measured using NIRS over the exposure and immersion times. Furthermore, the diffusion coefficient of the adhesives was calculated directly from the obtained moisture distribution in the adhesive layer by fitting it to a diffusion model.

## 2 Experimental Section

### 2.1 Adhesives

In this study, three types of adhesives were prepared to investigate the effects of curing agents and additives on moisture diffusion in the adhesive layers. Tables 1 and 2 present the chemical compositions of Adhesives I and II used in this study, respectively. These adhesives were used to examine the effects of curing agents on moisture diffusion. Both adhesives were composed of bisphenol A epoxy resin (jER 828; Mitsubishi Chemical Corp., Tokyo, Japan) and a curing agent. Generally, adhesives contain many additives, such as silica and fillers, in addition to resins and curing agents. However, when adhesives containing many additives are exposed to a humid/water environment, the diffusion process becomes complicated owing to the reduction in the free volume and the accumulation of moisture at the interface between the resin and the additives. Therefore, adhesives with simple compositions are recommended for investigating moisture diffusion.^27^ For Adhesive I, poly(propylene glycol) bis(2-aminopropyl ether) with an average Mn of approximately 230 (Sigma-Aldrich 406651, Merck KGaA, Darmstadt, Germany), which is a hydrophobic diamine, was used as the curing agent, and the curing conditions were set to room temperature (approximately 23 °C) for 1 week. For Adhesive II, Dicyandiamide (DICY; C0454, Tokyo Chemical Industry Co., Ltd., Tokyo, Japan) was used as a curing agent, and the curing condition was set to 180 °C for 30 min. Table 3 presents the chemical composition of Adhesive III, which was composed of the same resin and curing agent as those of Adhesive II. The effect of additives on moisture diffusion was investigated by comparing Adhesives II and III. Adhesive III, supplied by Cemedine Co., Ltd. (Tokyo, Japan), is a traditional structural epoxy adhesive that achieves a good balance of strength and toughness and contains silica, a filler, and carboxyl-terminated butadiene acrylonitrile (CTBN) rubber in addition to bisphenol A epoxy resin and DICY.^17,28−30^ The curing condition of Adhesive III was set to 180 °C for 60 min.

**Table 1:** Chemical Composition of Adhesive I

Material	Mass [%]
Bisphenol A epoxy resin	76
Poly(propylene glycol) bis(2-aminopropyl ether)	24

**Table 2:** Chemical Composition of Adhesive II

Material	Mass [%]
Bisphenol A epoxy resin	88
Dicyandiamide	10
3-(3,4-dichlorophenyl)-1,1′-dimethylurea	2

**Table 3:** Chemical Composition of Adhesive III

Material	Mass [%]
Bisphenol A epoxy resin	24
Carboxyl-terminated butadiene acrylonitrile rubber Modified epoxy resin (elastomer 40%)	39
Fumed silica	3
Filler (CaCO_3_)	26
CaO	2
Dicyandiamide	5
3-(3,4-dichlorophenyl)-1,1′-dimethylurea	1

### 2.2 Specimens

A quartz glass plate measuring 25 mm in length and width and 1 mm in thickness was used as the substrate. Because quartz glass allows high transmission in the NIR range and does not absorb moisture, it is suitable for investigating the moisture absorbed in adhesive layers using NIRS. The surface of the glass plate was degreased with acetone before bonding.

**Figure 1:**
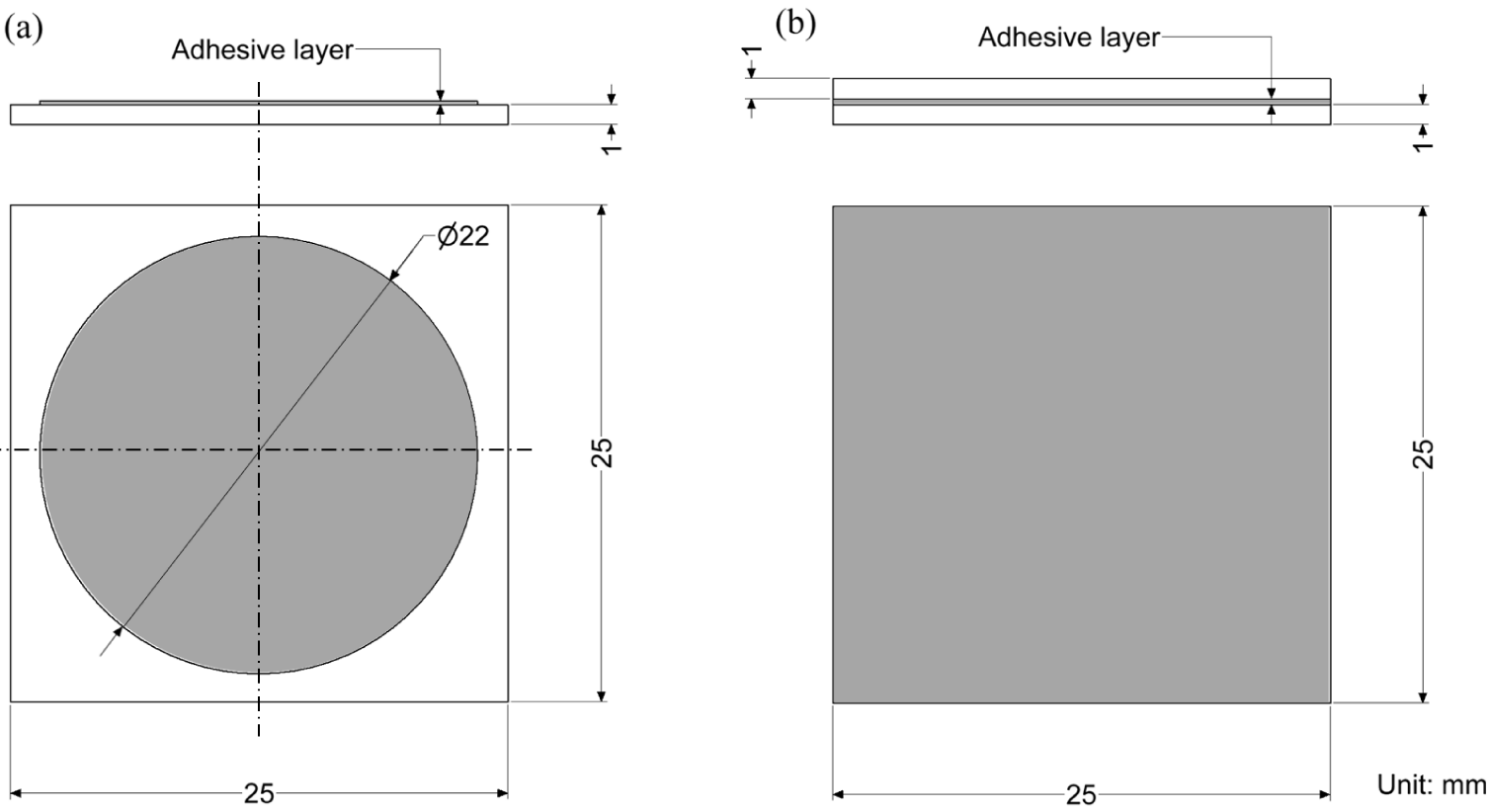
Schematics of (a) open-face and (b) closed specimens.

**Figure 2:**
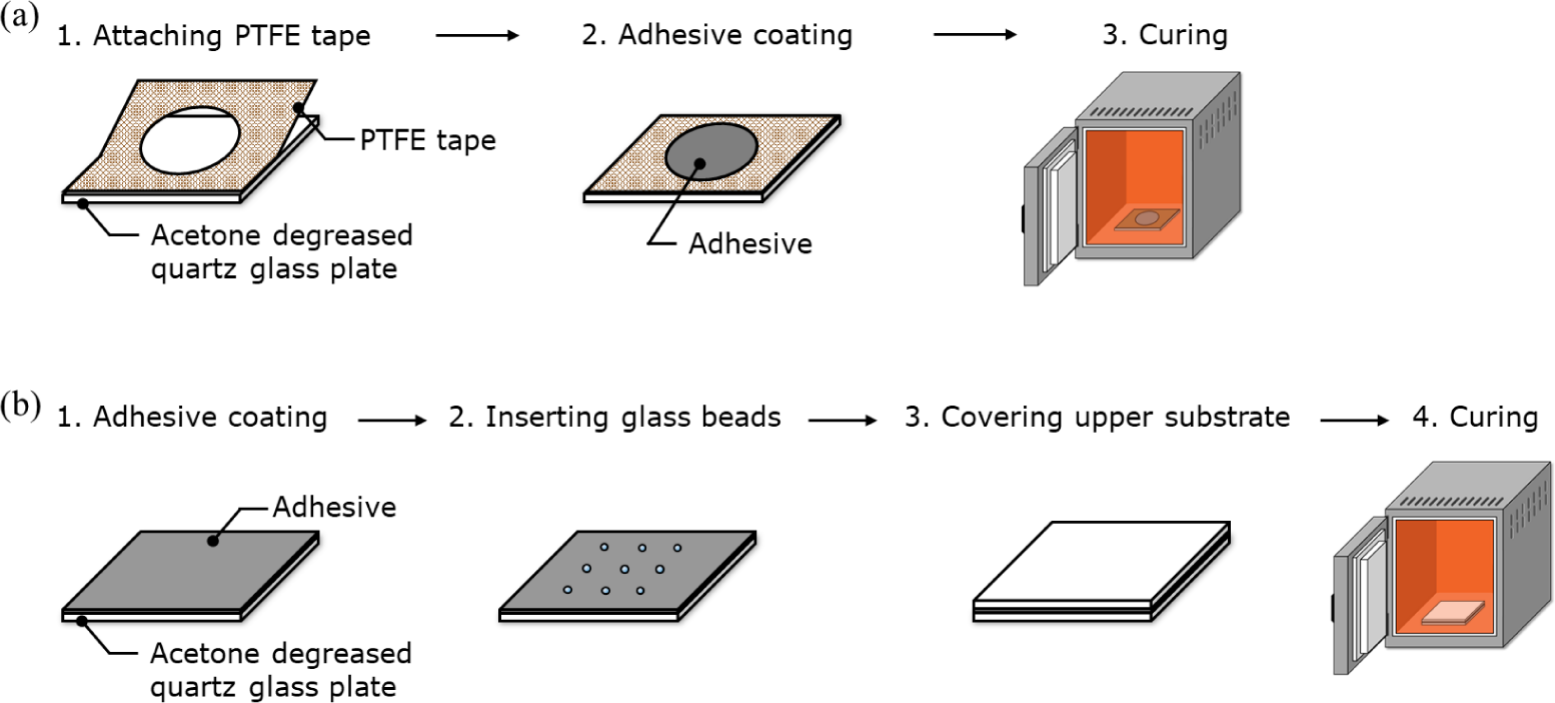
Specimen fabrication flowcharts for (a) open-face and (b) closed specimens.

Two types of specimens were prepared to investigate moisture diffusion in the adhesive layers: open-face and closed specimens. The schematics of both specimens are shown in Figure 1. For the open-face specimen, the adhesive was applied to the surface of the substrate, allowing rapid and uniform moisture diffusion into the adhesive. Open-face specimens were used for gravimetric and spectral analyses to determine the bulk diffusion coefficients. To control the thickness of the adhesive, a 0.18 mm polytetrafluoroethylene (PTFE) tape with a 22 mm diameter hole was attached to the quartz glass plate. The adhesion area was approximately 380 mm^2^, as shown in Figure 1a. The thickness of the adhesive was measured before testing. In closed specimens, the adhesive layer was sandwiched between the substrates, resulting in moisture diffusion from the edge to the center of the specimen. Therefore, closed specimens were used to measure moisture distribution in the adhesive layer. Adhesive thicknesses were controlled by inserting glass beads with a diameter of 0.3 mm prior to curing. The adhesion area was 625 mm^2^ and is shown in Figure 1b. A flowchart of the entire specimen fabrication process is shown in Figure 2.

### 2.3 Experimental Setup

**Figure 3:**
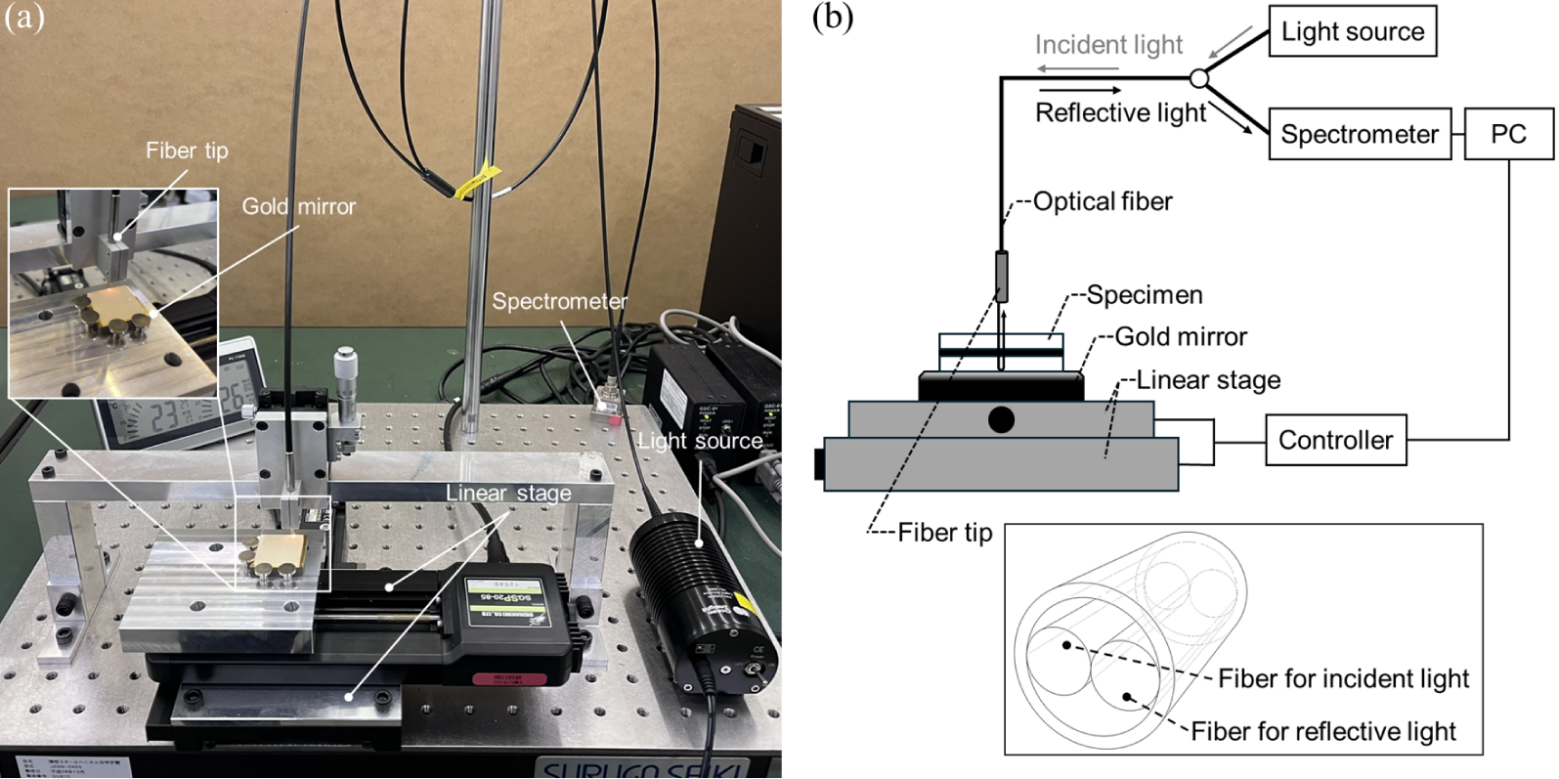
(a) Photograph of the NIRS measurement setup and (b) schematic illustration of the instrument operation.

The NIRS measurement setup is shown in Figure 3a. The system included a light source consisting of a tungsten-halogen lamp (HL-2000-LL, Ocean Insight, Orland, USA), an automatic x–y linear stage, an NIR spectrometer capable of examination in the wavelength range of 1750–2150 nm (NIRONE S2.2, Spectral Engines, Steinbach, Germany), and a bifurcated optical fiber bundle. Figure 3b shows a schematic of the instrument operation. Light from the light source irradiated the specimen along the optical fiber. Because quartz glass plates were used as the upper and lower substrates, a flat gold mirror (TFG-30S05–10, Sigmakoki Co., Ltd., Tokyo, Japan), which reflects more than 98% of the NIR rays, was placed under the specimen. The reflected light reentered the optical fiber and was detected using a spectrometer.

### 2.4 Experimental Procedure

**Figure 4:**
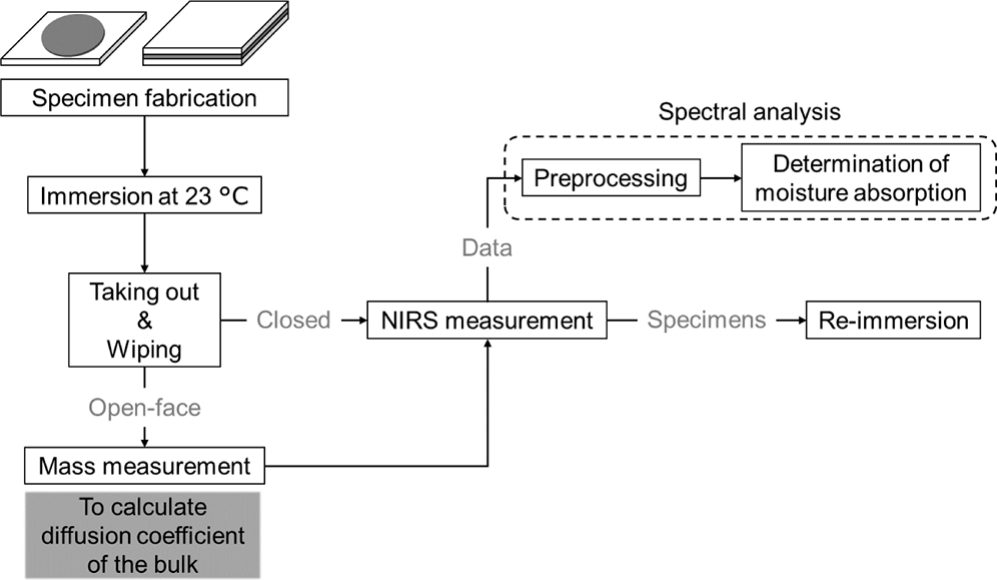
Experimental procedure from specimen fabrication to data analysis.

**Figure 5:**
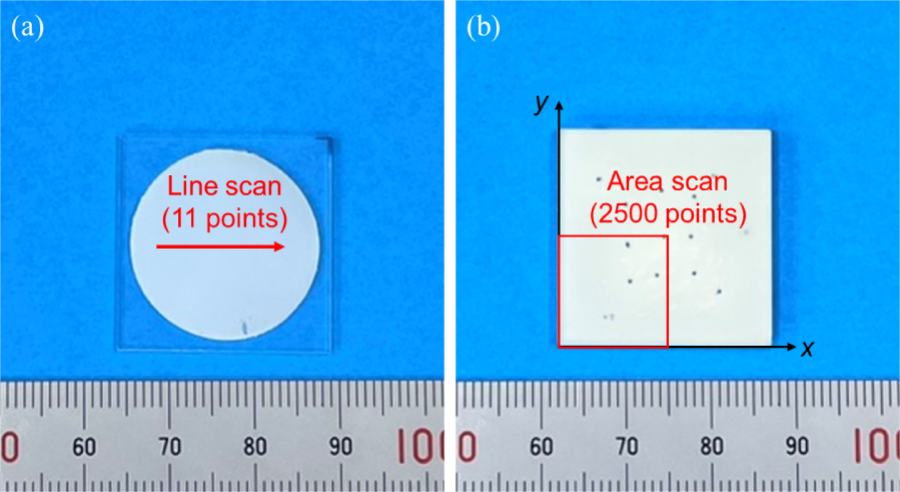
Measurement area of (a) open-face and (b) closed specimens.

The experimental procedure from specimen fabrication to data analysis is shown in Figure 4. First, the specimens were exposed to a humid environment in a desiccator, with the internal relative humidity controlled at 100% RH, or immersed in a glass bottle filled with purified water at room temperature. After the exposure or immersion, the specimen was wiped and installed on the measurement system. For the open-face specimens, 11 points along the centerline of the specimen were measured with a scanning pitch of 0.5 mm (see Figure 5a). The spectra of the open-face specimens were measured at 1 nm wavelength intervals. For the closed specimens, only a quarter of the specimen surface was measured considering symmetry, setting a scanning pitch of 0.25 mm and a wavelength interval of 5 nm. Therefore, 2500 points were scanned for each measurement. For the open-face specimens, the mass was also measured using an electronic balance (AP125WD, Shimadzu Co., Kyoto, Japan) with a resolution of 0.01 mg to investigate gravimetric changes. After the measurements were obtained, the specimens were returned to the experimental environment.

### 2.5 Spectral Analysis

The measured intensity spectra were converted to absorbances as follows:
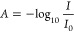
1where *I* is the intensity spectrum of the specimens and *I*_0_ is the background spectrum. A background spectrum was obtained prior to each measurement using a plane glass plate for open-face specimens and two glass plates with an air gap of the same thickness as that of the adhesive layer for closed specimens. When analyzing NIRS spectral peaks, spectrum shifts or baseline rotations become obstacles. Therefore, the acquired spectra must be preprocessed.^31−33^ The spectra acquired in this study were smoothed using a third-order Savitzky–Golay filter. This digital filter was designed to enhance data precision without distorting signal trends. Spectral smoothing was implemented using the scipy.signal.savgol_filter module in Python 3.10. Considering the trends in the data without distortion, the filter window was set to 60 for the open-face specimen and 9 for the closed specimen. Subsequently, the second-derivative technique was used to eliminate the shift and rotation effects.^32^ The peak derived from absorbed moisture in the absorbance spectra appeared in the negative direction in the second-derivative spectra. Therefore, NIRS moisture absorption was determined by the absolute value of the peak in the second-derivative spectra. For open-face specimens, the total NIRS moisture absorption was calculated using the average NIRS moisture absorption at 11 points. For closed specimens, NIRS moisture absorption values were plotted at each coordinate to visualize the moisture distribution in the adhesive layer.

## 3 Results and Discussion

### 3.1 Diffusion Coefficient of Bulk

#### 3.1.1 Effect of Curing Agents on Diffusion Behavior

**Figure 6:**
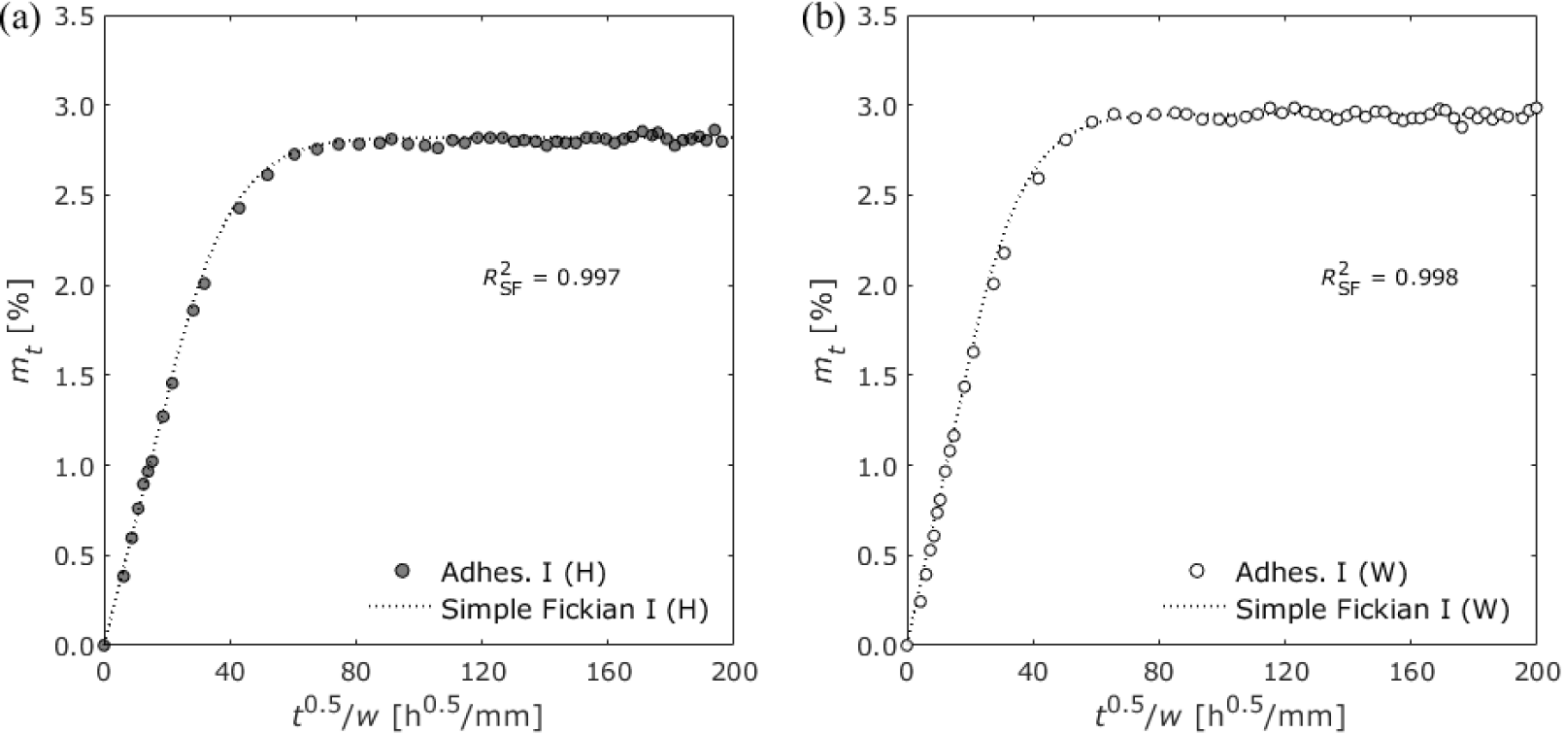
Gravimetric changes of Adhesive I exposed to (a) 100% RH and (b) water immersion.

**Figure 7:**
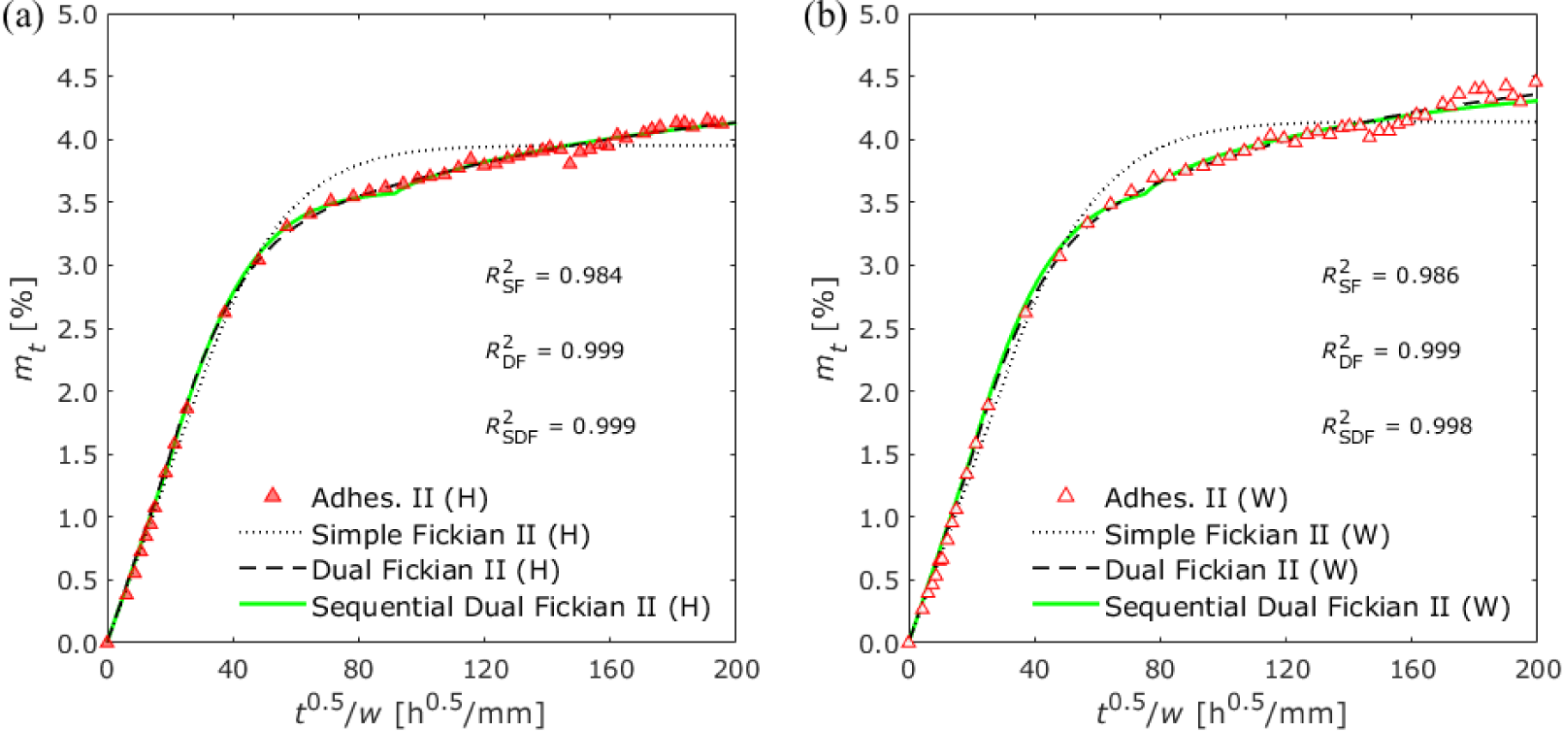
Gravimetric changes of Adhesive II exposed to (a) 100% RH and (b) water immersion.

**Figure 8:**
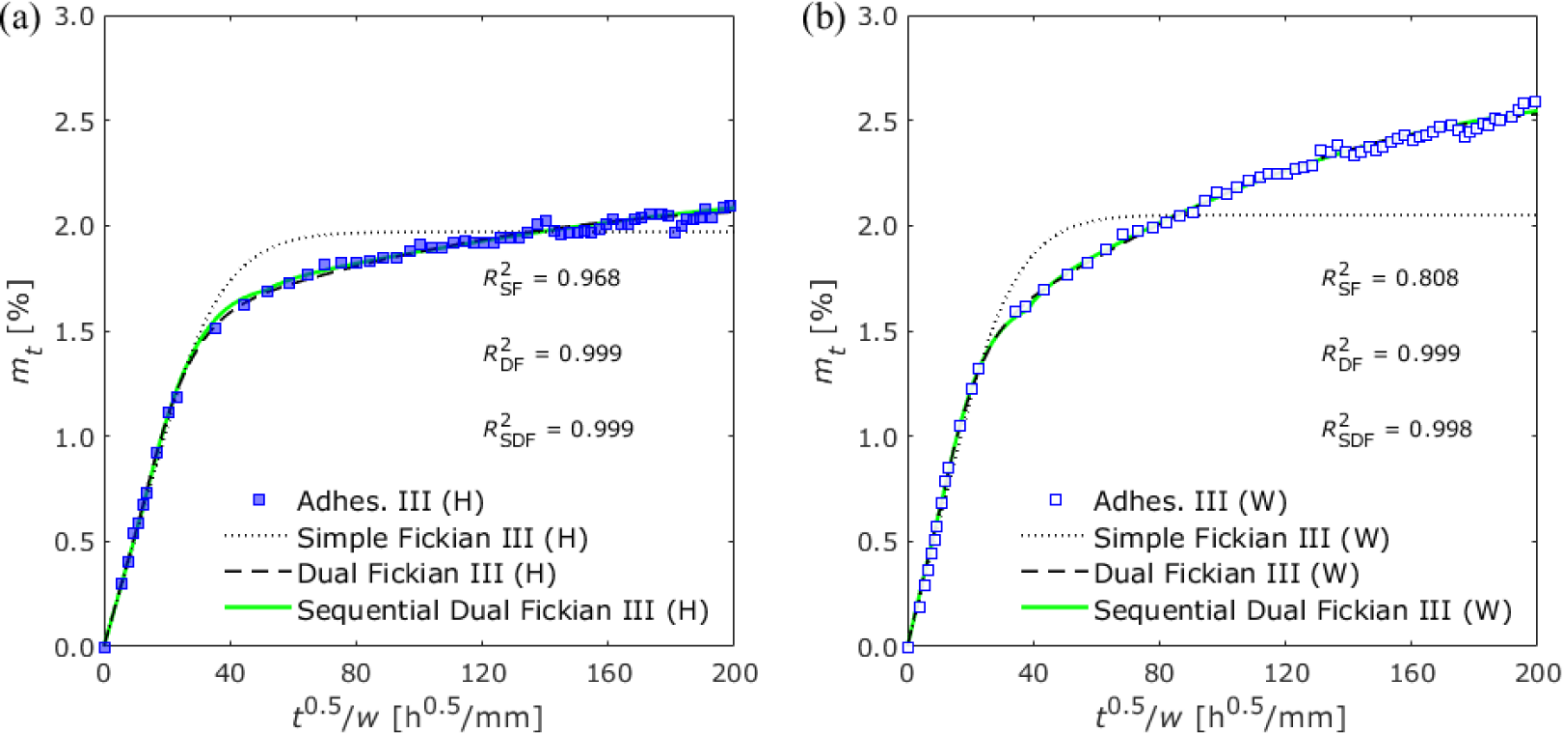
Gravimetric changes of Adhesive III exposed to (a) 100% RH and (b) water immersion.

A gravimetric method using open-face specimens was employed to determine the diffusion coefficients of the adhesives. The gravimetric method is a general method used for measuring the amount of absorbed moisture using the weight change as follows:
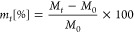
2where *m*_*t*_ is the moisture content, *M*_0_ is the initial mass of the adhesive, and *M*_*t*_ is the mass of the adhesive at time *t*. The mass of the quartz glass plate measured before specimen fabrication was subtracted from the total mass of the specimen to calculate the mass change of the adhesive. Gravimetric changes in Adhesive I, II, and III under high-humidity exposure and water immersion conditions are shown in Figures 6, 7, and 8, respectively.

Simple Fickian (SF) diffusion is a classical theory based on Fick’s second law, which considers only the diffusion of water molecules into the free volume of the system.^34,35^ For one-dimensional analysis, the moisture content at time *t* is expressed as follows:^21^
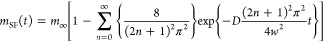
3where *m*_∞_ is the saturated moisture content, *w* is the adhesive thickness, and *D* is the diffusion coefficient. Equation 3 can be approximated as
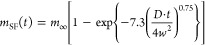
4

The adhesive thicknesses of the open-face specimens are listed in Table 4. By substituting the adhesive thickness into eq 4 and fitting it to the gravimetric changes for each adhesive using the least-square method, *m*_∞_ and *D* were obtained and are presented in Table 5. Adhesive I, which used a hydrophobic diamine as a curing agent, showed good agreement with simple Fickian diffusion (Figure 6). When the bisphenol A epoxy resin was cured using poly(propylene glycol) bis(2-aminopropyl ether), flexible and long bonds were formed.^36^ Owing to the sufficient free volume provided by the long and flexible bonds, the diffusion process in Adhesive I could be regarded as governed by free volume diffusion. Conversely, in the case of Adhesives II and III, that is, when DICY was used as the curing agent, the moisture content continued to increase even after the inflection point appeared, as shown in Figures 7 and 8. Therefore, simple Fickian diffusion does not always adequately explain the diffusion process in epoxy adhesives, resulting in the misestimation of diffusion or failure to predict the second stage of diffusion.^37,38^ In addition to free volume diffusion, the relaxation of the polymer system can influence the diffusion process, known as anomalous diffusion.^21^ In particular, curing agents with strong hydrophilicity, such as DICY, tend to trap water molecules in the polymer chain via hydrogen bonds.^39^ This bound water can induce polymer swelling and loss, potentially facilitating anomalous diffusion behavior.^40,41^ As most initial diffusion behaviors are Fickian diffusion in anomalous diffusion, the relaxation rate and chemical bonds are negligible. As the absorbed moisture increases, more water molecules bond to the polymer chain, limiting the amount of moisture that can be absorbed by classical diffusion by restricting the space within the nanopores. Subsequently, the relaxation rate became larger than the diffusion rate, governing the remaining diffusion process.^37^

**Table 4:** Adhesive Thickness of the Open-Face Specimens Used in this Study

Specimen type	Environment	Adhesive type	Adhesive thickness (*w*) [mm]
Open-face	Humid	I	0.160
		II	0.161
		III	0.181
	Water	I	0.164
		II	0.162
		III	0.185

**Table 5:** Results of Fitting Simple Fickian Diffusion to the Gravimetric Changes of Each Adhesive

Environment	Adhesive	*m*_∞_ [%]	*D* [mm^2^/h]	*R*^2^
100% RH	I	2.82	4.15 × 10^–4^	0.997
	II	3.95	2.14 × 10^–4^	0.984
	III	1.97	4.68 × 10^–4^	0.968
Water immersion	I	2.95	5.65 × 10^–4^	0.998
	II	4.14	1.91 × 10^–4^	0.986
	III	2.05	5.65 × 10^–4^	0.808

Various models have been proposed to explain anomalous diffusion, including dual Fickian (DF) diffusion, based on two Fickian diffusion processes.^14,20,42,43^ The moisture content of a dual Fickian diffusion model is expressed as
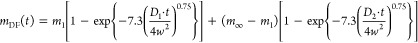
5where *m*_1_ and *m*_∞_ are the saturated moisture content at the first diffusion stage and the total diffusion stage, respectively. The diffusion coefficients of the first and second diffusion stages are *D*_1_ and *D*_2_. The dual Fickian diffusion model assumes that two different mechanisms occur simultaneously. Therefore, the moisture content increases continuously to reach the final saturation. Ameli et al.^20^ proposed a sequential dual Fickian (SDF) diffusion model by considering the possibility of the existence of a pseudoequilibrium state during the intermediate exposure time before reaching the final saturation. Thus, the two simple Fickian diffusion processes are operated sequentially. The moisture content in the sequential dual Fickian diffusion is expressed as
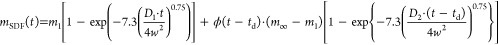
6where *t*_d_ is the delay time to start the second stage of diffusion, and ϕ(*t*) is the Heaviside step function and is defined as follows:
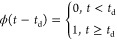
7

Substituting the adhesive thickness into eqs. 5 and 6 and fitting them to the gravimetric changes for Adhesives II and III, the moisture contents and diffusion coefficients were obtained, and the results are presented in Tables 6 and 7, respectively. Both the dual Fickian and sequential dual Fickian diffusion models showed good agreement with gravimetric changes in Adhesives II and III, as shown in Figures 7 and 8. The diffusion coefficients of the two models were found to be similar.

**Table 6:** Results of Fitting Dual Fickian Diffusion to the Gravimetric Changes of Each Adhesive

Environment	Adhesive	*m*_1_ [%]	*m*_∞_ [%]	*D*_1_ [mm^2^/h]	*D*_2_ [mm^2^/h]	*R*^2^
100% RH	II	3.13	4.33	3.65 × 10^–4^	1.56 × 10^–5^	0.999
	III	1.53	2.11	8.38 × 10^–4^	2.48 × 10^–5^	0.999
Water immersion	II	3.11	4.54	3.50 × 10^–4^	1.85 × 10^–5^	0.999
	III	1.44	2.62	1.19 × 10^–3^	2.50 × 10^–5^	0.999

**Table 7:** Results of Fitting Sequential Dual Fickian Diffusion to the Gravimetric Changes of Each Adhesive

Environment	Adhesive	*m*_1_ [%]	*m*_∞_ [%]	*D*_1_ [mm^2^/h]	*D*_2_ [mm^2^/h]	*t*_*d*_[h]	*R*^2^
100% RH	II	3.59	4.34	3.00 × 10^–4^	1.24 × 10^–5^	219	0.999
	III	1.72	2.24	7.03 × 10^–4^	9.78 × 10^–6^	87.8	0.998
Water immersion	II	3.64	4.45	3.05 × 10^–4^	1.71 × 10^–5^	149	0.999
	III	1.65	2.68	1.03 × 10^–3^	1.89 × 10^–5^	50.9	0.998

#### 3.1.2 Effect of Additives on Diffusion Behavior

Adhesive II showed a higher magnitude of moisture content than Adhesive III. Conversely, the first-stage diffusion coefficients of Adhesive III were significantly larger than those of Adhesive II. During the second stage of diffusion, no significant difference was observed between the two adhesives. Therefore, when the resin contains additives, the moisture initially penetrates faster, but the total amount is lower.

Adhesive III contained a filler, silica, and CTBN in addition to the resin and curing agent, such additives make a difference. For example, water molecules are attracted to calcium oxide and quickly form calcium hydroxide. This reactivity with water can increase the diffusion rate.^44^ Previous studies have suggested that moisture accumulates at the interface between the resin and the additives, resulting in a continuous mass increase and relaxation near the interface.^27^ However, the effects of interface-related phenomena could not be evaluated based on the results of this study. Instead, the proportion of epoxy resin was reduced by the addition of additives, resulting in a reduction in the total amount of water absorption.

#### 3.1.3 Effect of Exposure Conditions on Diffusion Behavior

By comparing water immersion tests with 100% RH exposure tests, the effect of the state of H_2_O (air or liquid) on the boundary conditions for moisture penetration can be investigated. However, care must be taken to prevent condensation from forming on the sample surface for 100% RH exposure tests. The test temperature was room temperature, and there was no temperature difference when the test specimen was inserted into or removed from the humidity-controlled chamber, so no water droplets were observed on the surface of the test specimens, in this study.

For Adhesives I and II, with simple compositions, the saturated moisture content upon water immersion was slightly higher than that upon 100% RH exposure. For Adhesive III with additives, this increase was more pronounced, increasing by more than 20%.

Fick’s law assumes that the driving force of diffusion is the concentration gradient. However, because diffusion is an intrinsic property of systems following equilibrium, the primary driving force is the chemical potential gradient.^45,46^ When water is in equilibrium in its vapor and liquid states, such as at 100% RH, the chemical potential should be the same. Hence, the influence of the exposure environment on diffusion was the same for 100% RH exposure and water immersion.^46^ However, as the moisture content approached saturation, the specimen immersed in water absorbed more water molecules than that exposed to 100% RH. Therefore, factors other than diffusion boundary conditions may be responsible for this deviation. If the adhesive contains soluble components, such as uncured components, it may leach into the water. A previous study suggested that the voids formed by leaching could influence the increase in the saturated moisture content.^12^ In particular, when additives are included, the possibility of leaching increases.

### 3.2 Spectral Analysis of Materials

**Figure 9:**
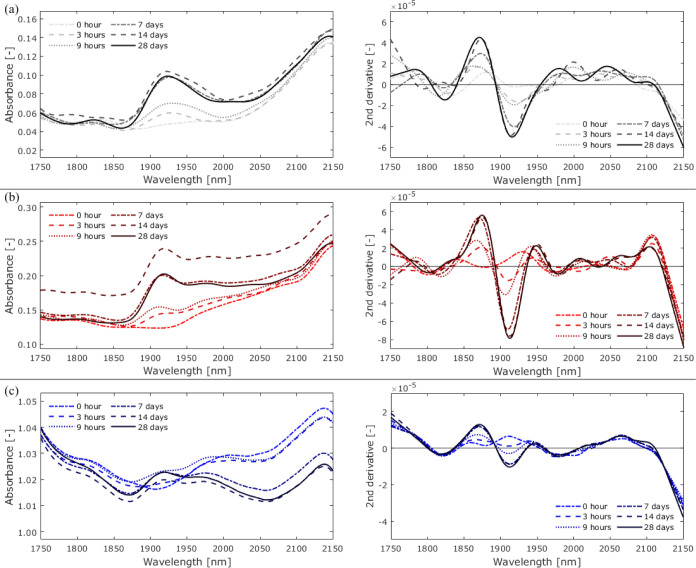
Changes in absorbance and the second-derivative spectra of (a) Adhesive I, (b) Adhesive II, and (c) Adhesive III exposed to 100% RH from 0 h (before exposure) to 28 days.

**Figure 10:**
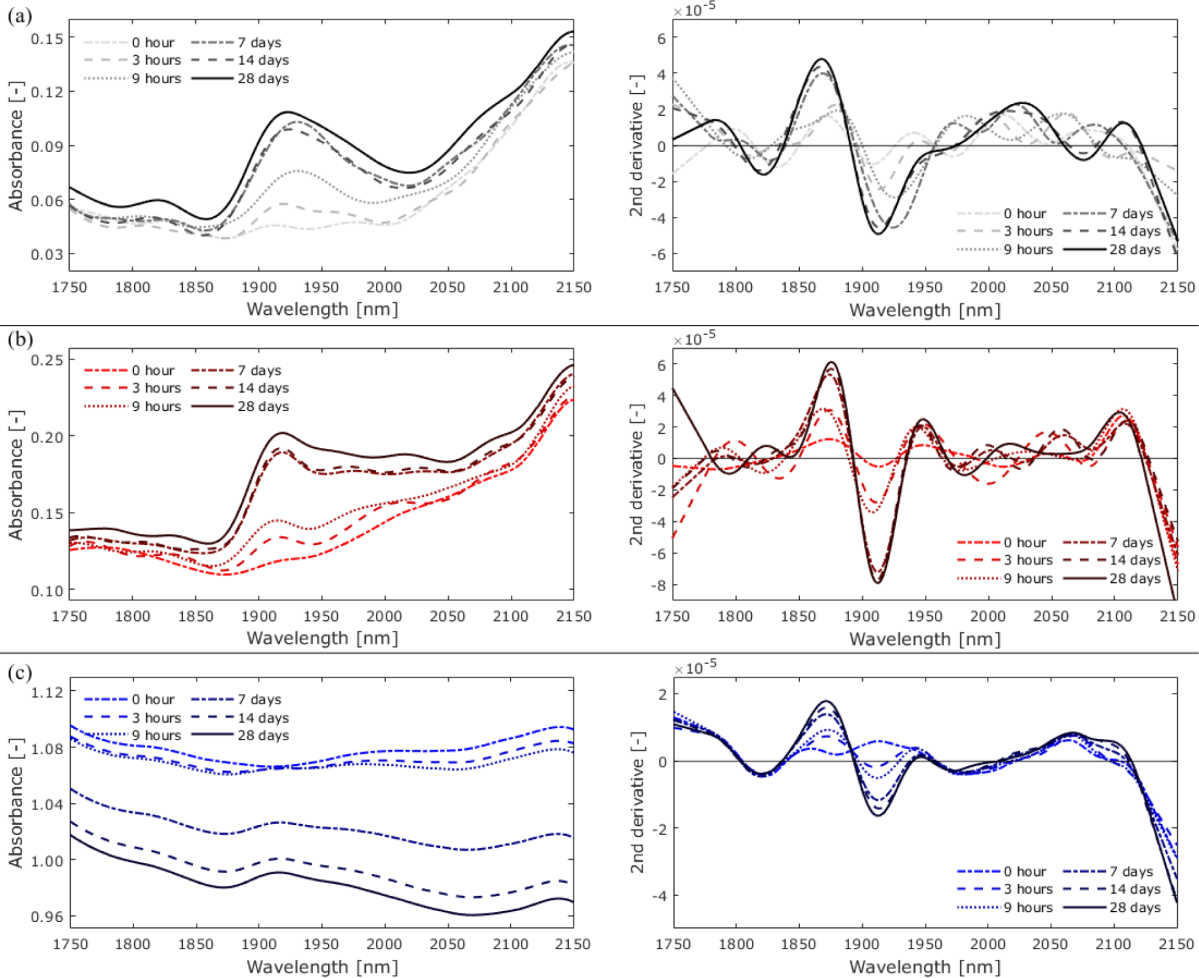
Changes in absorbance and the second-derivative spectra of (a) Adhesive I, (b) Adhesive II, and (c) Adhesive III immersed in water from 0 h (before exposure) to 28 days.

**Figure 11:**
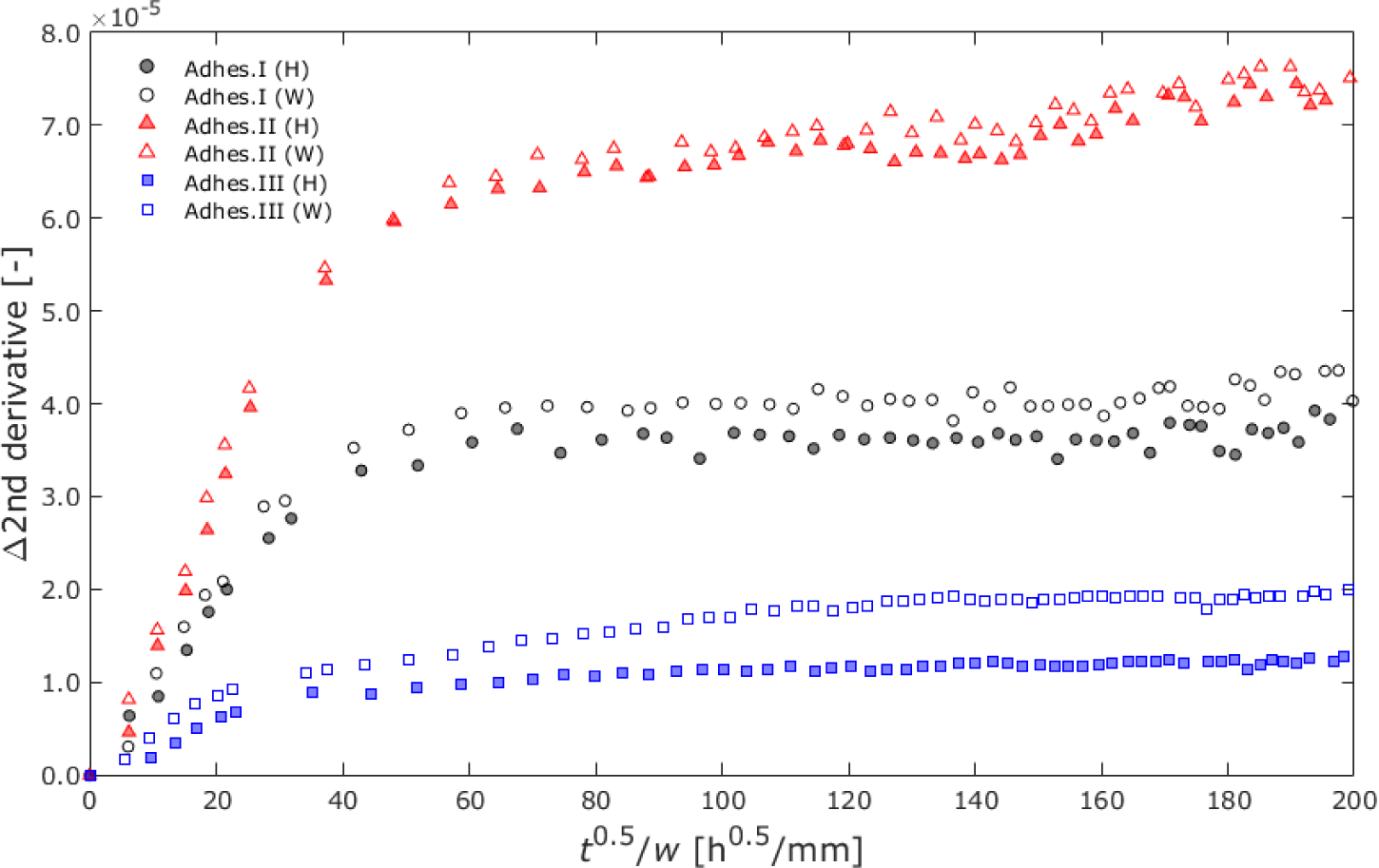
NIRS absorption changes of each adhesive exposed to 100% RH and water immersion.

**Figure 12:**
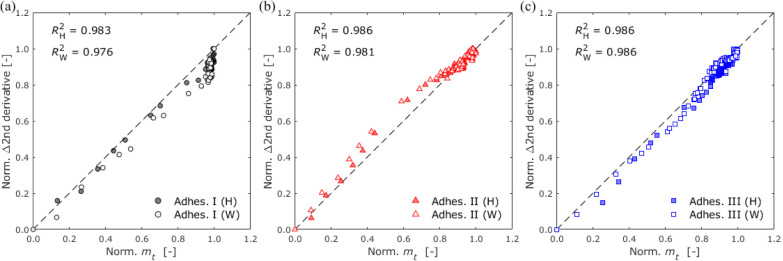
Comparisons of normalized gravimetric changes and NIRS absorption changes for moisture content in (a) Adhesive I, (b) Adhesive II, and (c) Adhesive III.

Figures 9 and 10 show the spectral changes observed in open-face specimens exposed to high humidity and water immersion, respectively. These spectral changes were measured for 28 days. The absorbance band of moisture exists in the NIR range of 1900–1950 nm.^47,48^ A peak increase with increasing exposure time was observed near 1920 nm. Because the spectral shift and rotation of the measured absorbance spectra must be removed using the second-derivative technique,^17,32^ the moisture absorption assessment using NIRS was determined as the change in the absolute value of the peak in the second-derivative spectra from the value on day 0, as shown in Figure 11. For all adhesives, the NIRS absorption increased linearly with the square root of time during the initial exposure period, after which the rate of increment decreased. These trends were similar to those of the gravimetric changes. The absolute amount of absorbed moisture affects the absorbance intensity. Therefore, the second-derivative absorbance change becomes larger with larger *m*_∞_ When additives are included in the resin, the light in the adhesive is scattered by the additives, and the intensity of the reflected light decreases.^49^ Therefore, the absorbance intensity is not necessarily proportional to the amount of moisture absorption. However, the tendency of water immersion to show higher values than the exposure to high humidity is consistent with the gravimetric method. Furthermore, the gravimetric and absorbance intensity changes showed a good correlation when the data were normalized using the min–max normalization method, as shown in Figure 12.

### 3.3 Diffusion Coefficient Determination in Adhesive Layers

#### 3.3.1 Moisture Distribution in Adhesive Layers

**Figure 13:**
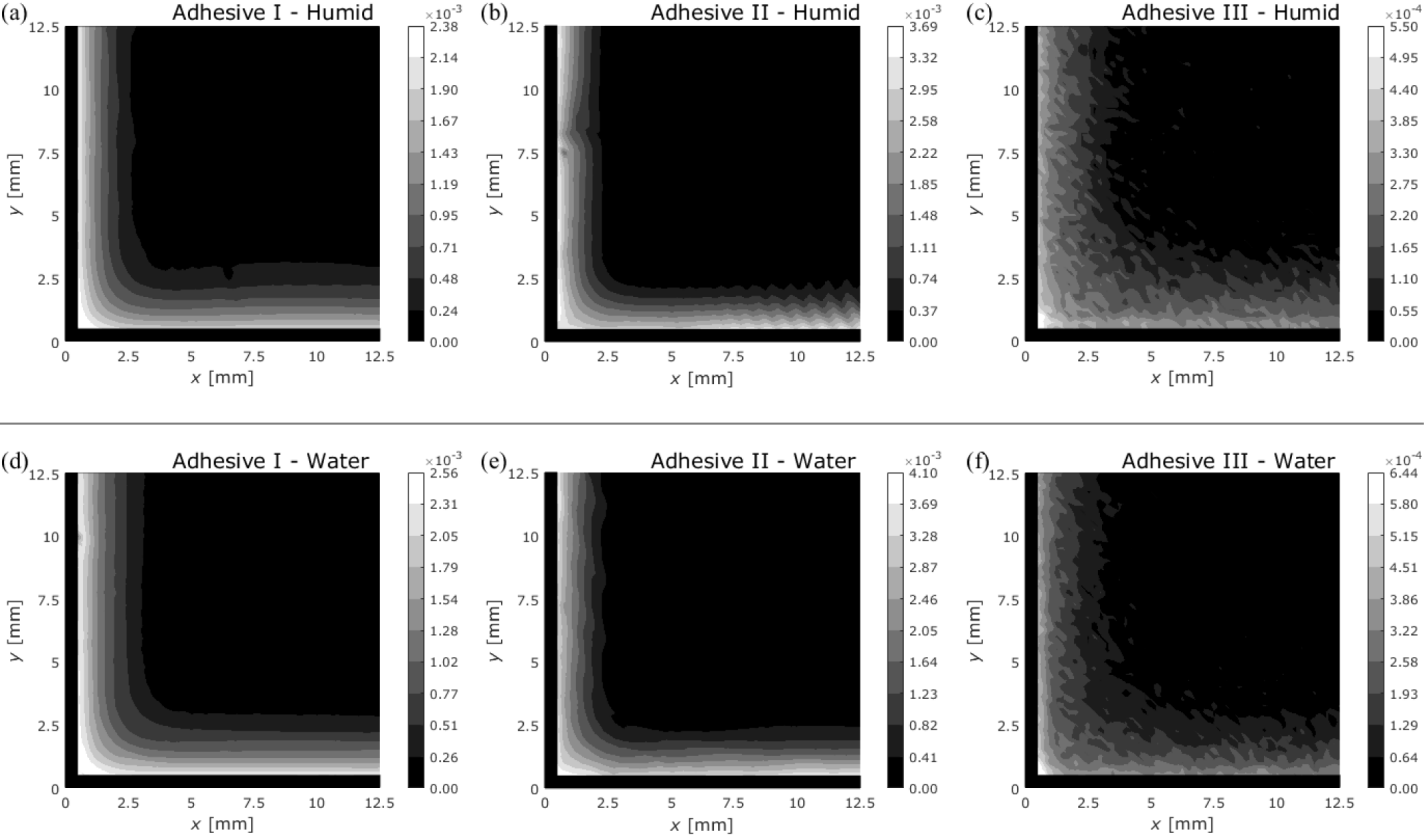
Moisture distribution of closed specimens with different exposure conditions: (a–c) 100% RH and (d–f) water immersion at an exposure time of 12 weeks. (a,d) Adhesive I; (b,e) Adhesive II; (c,f) Adhesive III. The maximum value of the contour is determined to be the maximum NIRS absorption of each distribution.

**Figure 14:**
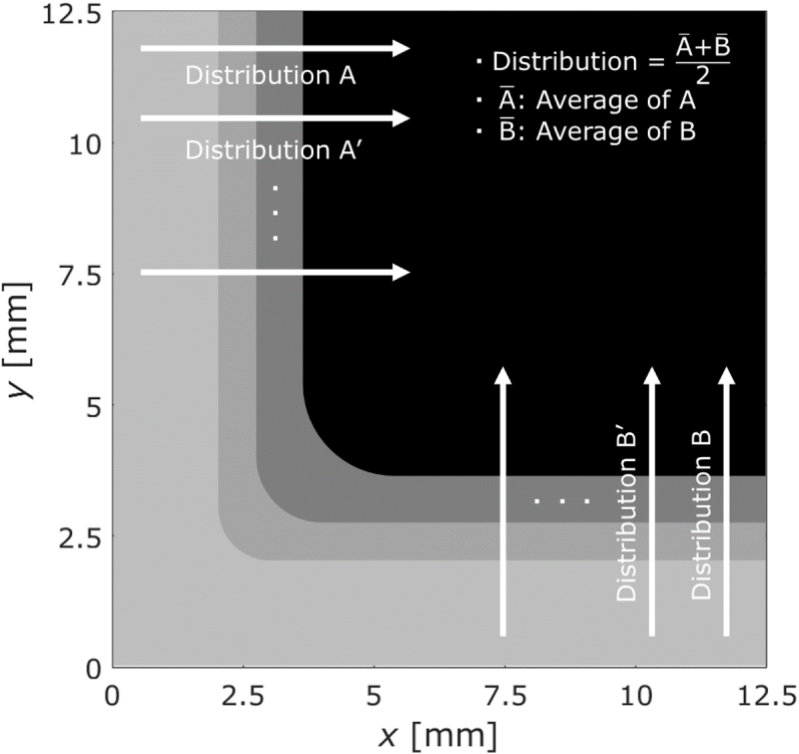
Schematic of the distances used in the analysis to determine the moisture distribution.

**Figure 15:**
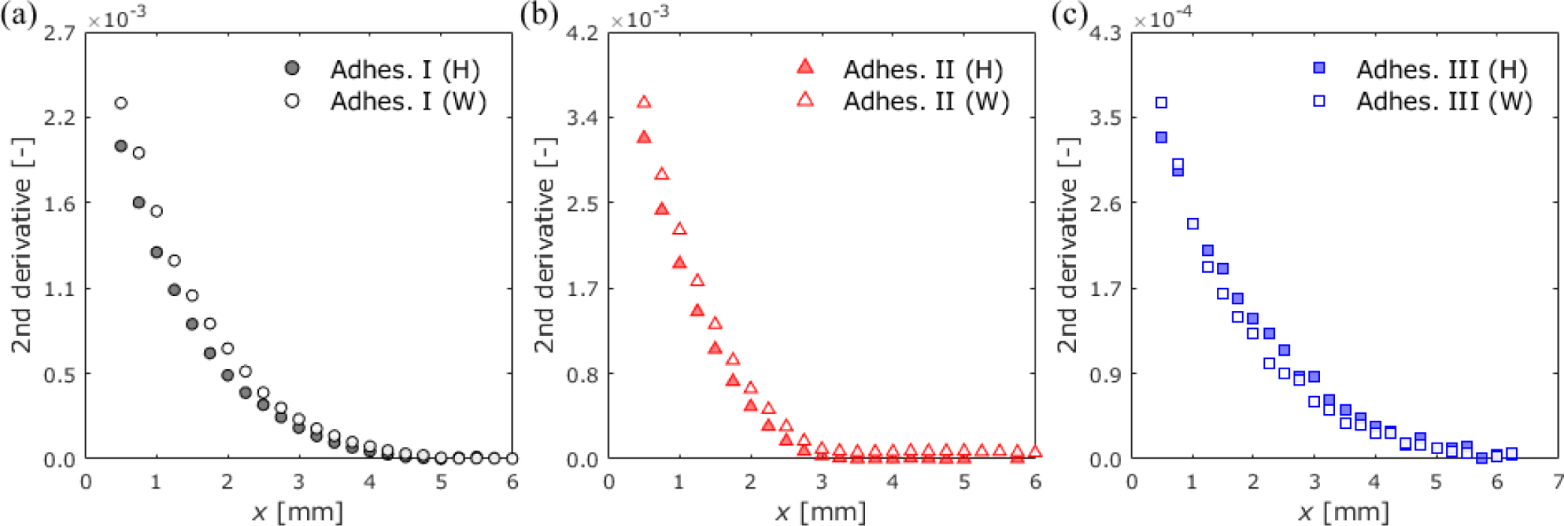
Moisture distribution in the adhesive layer of adhesives (a) I, (b) II, and (c) III as the relationship between the second-derivative intensity and the displacement from the edge at an exposure time of 12 weeks.

Figure 13 shows the moisture distribution of the closed specimens with each adhesive after 12 weeks of exposure. The region from 0 to 0.5 mm is represented in black because the scattering of light at the edges of the specimens prevented the acquisition of accurate moisture absorption data. When the moisture content is below the measurement resolution such as thin diffusion layer at the interface and adsorbed moisture on the surface of quartz glass plate, the NIRS absorption may show negative values; however, because there was no physical reason for this, the negative values were ignored. Nonuniform distributions were observed in Adhesive III compared with those in Adhesives I and II, as shown in Figure 13c,f. Light scattering by the additives might have reduced the peak intensity, lowered the maximum intensity, and resulted in noisy measurements. As the diffusion front from the side was not a perfectly straight line, the average value of the moisture distributions from 7.5 to 12.5 mm in both the x- and *y*-axis directions was adopted as the moisture distribution of the adhesive layer along the length, as shown in Figure 14. Figure 15 shows the moisture distributions after 12 weeks of exposure, where the *y*-axis represents the second-derivative intensity of the absorbance spectra. In calculating the diffusion coefficient, the *y*-axis needs to be converted to the moisture content. Therefore, the intensity needs to be normalized to the saturated intensity. However, light scattering makes it difficult to accurately measure the intensity of the edge area, where the intensity is the greatest. In addition, because the adhesive layer has a much greater distance for moisture diffusion than the bulk, waiting until the moisture content becomes saturated is impractical. Therefore, the experimental results cannot be directly converted to moisture content on the -axis. Here, the saturated intensity, that is, the boundary condition, was considered as an unknown value, and the unknown parameters were optimized such that the coefficient of determination between the distribution function and the experimental value approached 1.

#### 3.3.2 Simple Fickian Diffusion

Generally, moisture distribution in adhesive layers is predicted by substituting the diffusion coefficient into
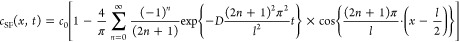
8which was derived from Fick’s second law, where *c*_SF_ is the moisture concentration at position *x* at time *t*, *c*_0_ is the boundary concentration, and *l* is the length of the closed specimen. The boundary condition was set in the range of 1–100% for high-humidity exposure and 100% for water immersion, and *l* was set to 25 mm.

**Figure 16:**
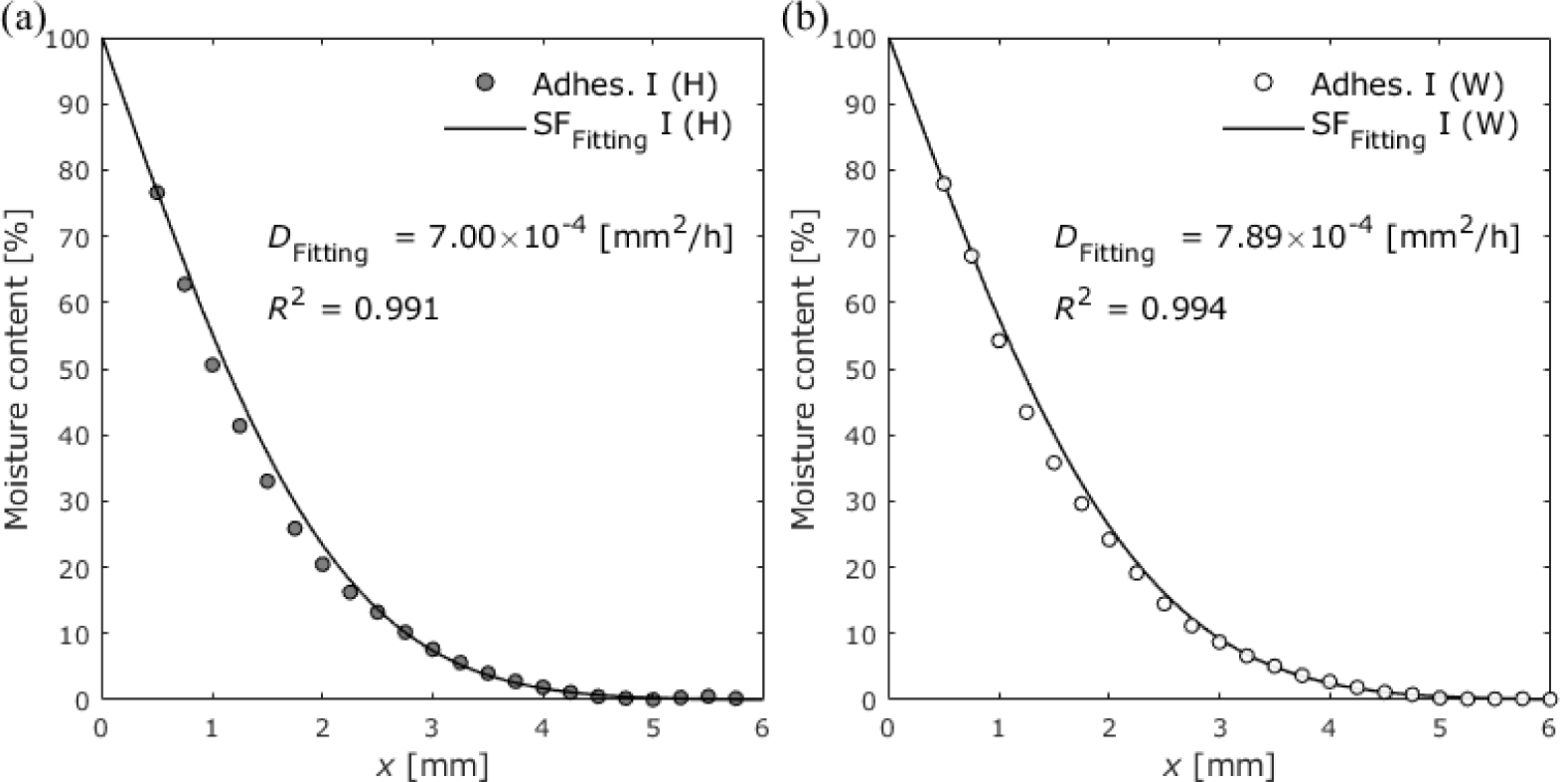
Average moisture distributions in closed specimens with Adhesive I at an exposure time of 12 weeks for (a) 100% RH exposure and (b) water immersion.

Because Adhesive I showed almost simple Fickian diffusion behavior, the diffusion coefficients in the adhesive layer were determined by fitting eq. 8 to the average moisture distribution for each period. The values of the fitted parameters from exposure times of 1 to 12 weeks were then averaged. The unknown parameters determined by fitting are the diffusion coefficient and boundary condition. The obtained boundary condition for high-humidity exposure was 100%. Table 8 shows the obtained diffusion coefficients compared with those in the bulk. Figure 16 shows the moisture distributions determined by data fitting after 12 weeks of exposure.

**Table 8:** Diffusion Coefficients of the Bulk and Adhesive Layer in Adhesive I Determined by Simple Fickian Diffusion

	Diffusion coefficient (*D* [mm^2^/h])	
Environment	Open-face	Closed (average)
100% RH	4.15 × 10^–4^	7.00 × 10^–4^
Water immersion	5.09 × 10^–4^	7.89 × 10^–4^

#### 3.3.3 Dual Stage Diffusion

For Adhesives II and III, the dual-stage diffusion models were more appropriate than the single-stage diffusion models. Therefore, eq 8 was modified by considering dual Fickian and sequential dual Fickian diffusion.

**Figure 17:**
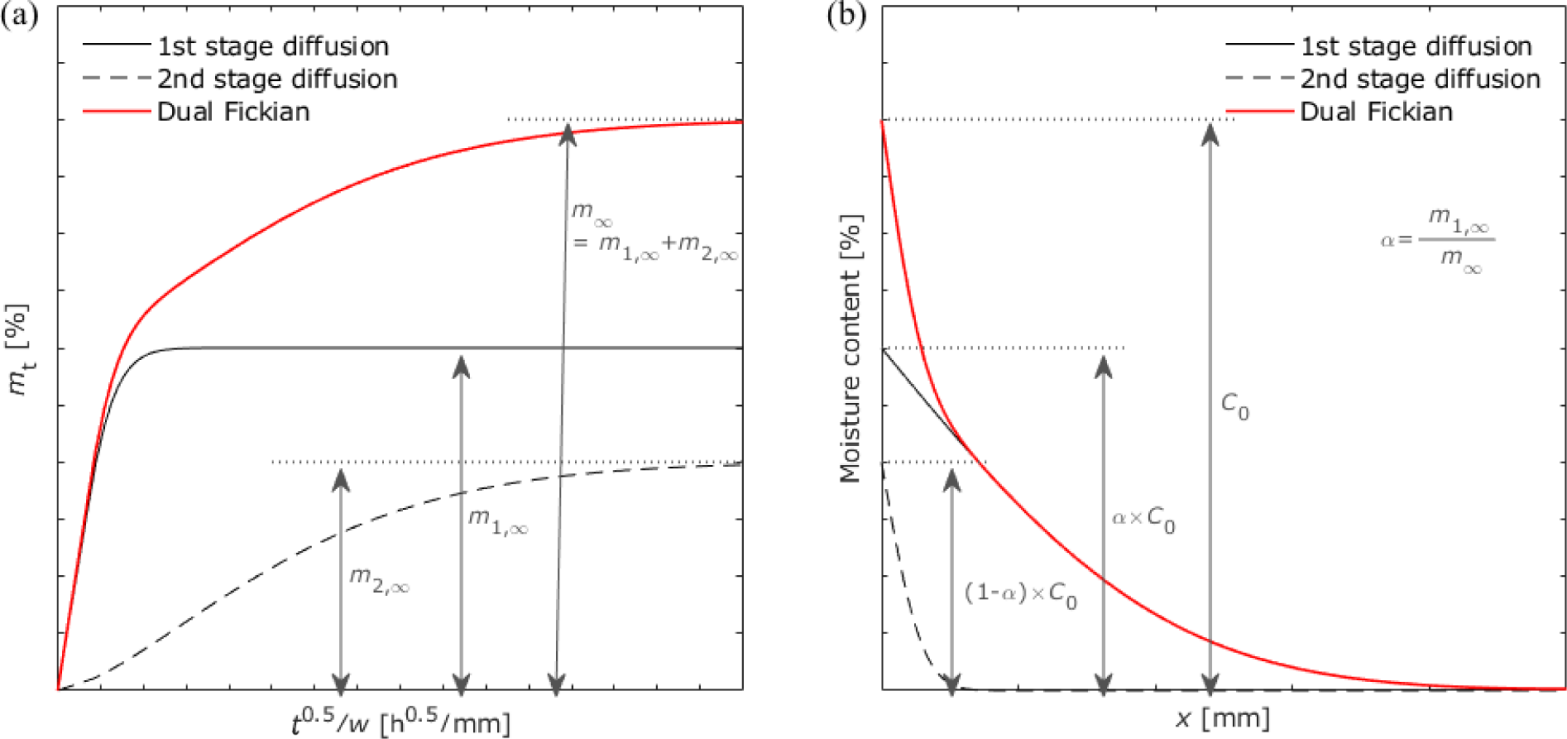
Schematic of the dual Fickian diffusion process: (a) gravimetric changes and (b) moisture distribution.

Figure 17a shows the change in moisture content over time in dual Fickian diffusion for the bulk. Two diffusion processes with different diffusion coefficients occurred simultaneously; however, the second stage of diffusion required a longer time to saturate. The moisture distribution for each diffusion process can be calculated using eq 8. Therefore, the total moisture distribution can be expressed as the sum of the two values as follows:
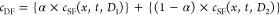
9where α is calculated by the ratio of the first stage to the entire diffusion as *α* = *m*_1_/*m*_∞_. Because the moisture associated with the first stage of diffusion penetrates faster, the central part of the adhesive layer was affected only by the first stage of diffusion, whereas the area near the edge is affected by both stages (see Figure 17b).

The average diffusion coefficients of Adhesives II and III were determined by fitting eq 9 in the same manner as that for Adhesive I. The unknown parameters were *D*_1_, *D*_2_, α, and the boundary condition. However, to reduce the fitting parameters, α obtained in the previous experiments for open-face specimens was employed. Thus, three parameters were fitted. Similar to the results of Adhesive I, the fitting results revealed a boundary condition of 100% under high humidity. Table 9 presents the obtained diffusion coefficients compared with those in the bulk.

**Table 9:** Diffusion Coefficients of the Bulk and Adhesive Layer in Adhesives II and III Determined by Dual Fickian Diffusion

		Diffusion coefficient (*D* [mm^2^/h])			
		Open-face		Closed (average)	
Adhesive	Environment	*D*_1_	*D*_2_	*D*_1_	*D*_2_
II	100% RH	3.65 × 10^–4^	1.56 × 10^–5^	4.03 × 10^–4^	2.22 × 10^–5^
	Water immersion	3.50 × 10^–4^	1.85 × 10^–5^	4.61 × 10^–4^	2.07 × 10^–5^
III	100% RH	8.38 × 10^–4^	2.48 × 10^–5^	1.53 × 10^–3^	2.03 × 10^–5^
	Water immersion	1.19 × 10^–3^	2.50 × 10^–5^	1.18 × 10^–3^	5.25 × 10^–5^

**Figure 18:**
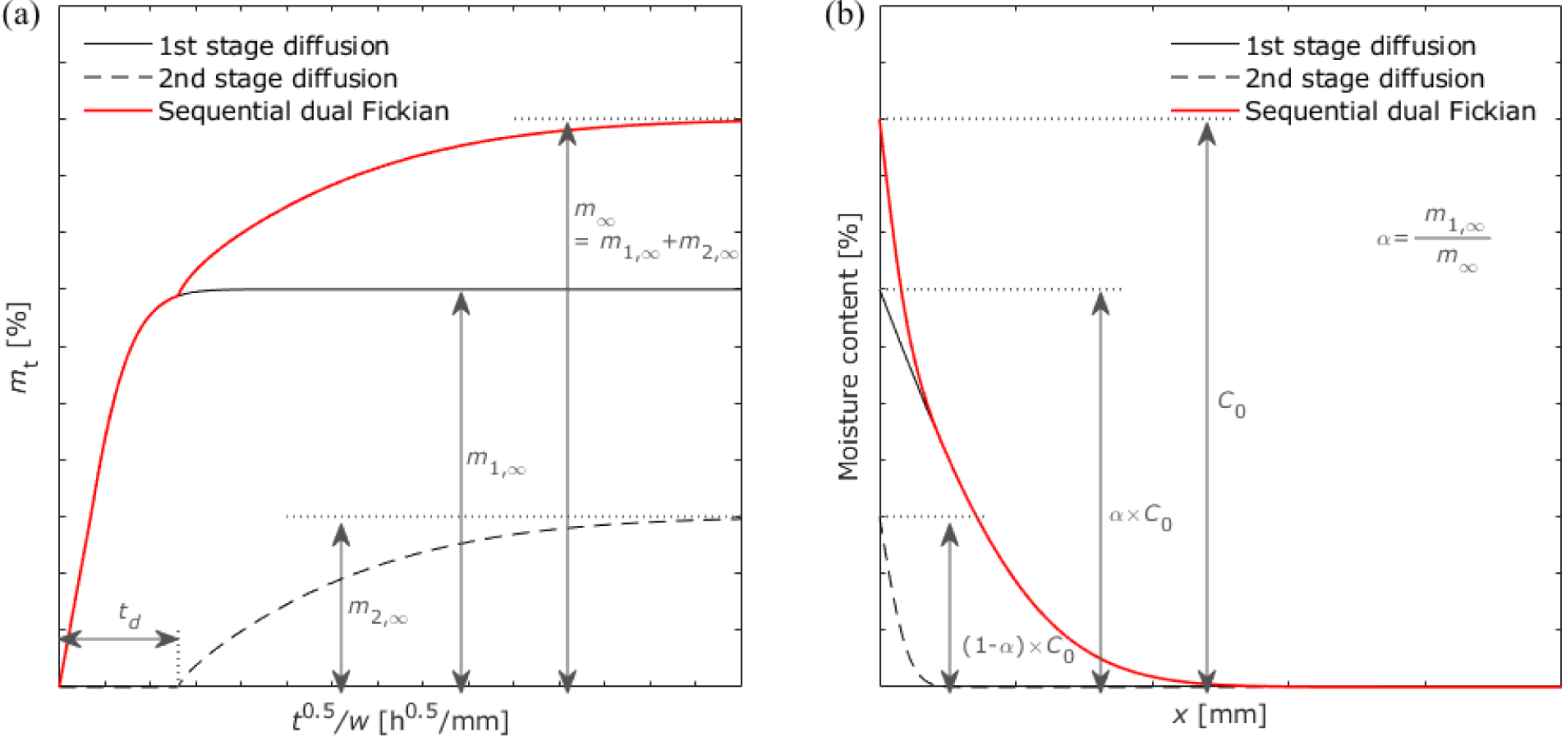
Schematic of the sequential dual Fickian diffusion process: (a) gravimetric changes and (b) moisture distribution.

Figure 18a shows the change in moisture content over time in sequential dual Fickian diffusion in the bulk. Two diffusion events with different diffusion coefficients occurred during these stages. Therefore, simply summing the moisture content of each diffusion process is insufficient, and the time delay must also be considered. The total moisture content can be expressed as follows:
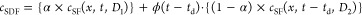
10where *t*_d_ is the delay time required to begin the second stage of diffusion. The overall shape of the moisture distribution in the adhesive layer was similar to that of the dual Fickian diffusion, as shown in Figure 18b. However, a slight difference was observed in the distribution at the edge area affected by both diffusion stages. The unknown parameters were *D*_1_, *D*_2_, α, *t*_d_, and the boundary condition. Similar to the fitting of dual Fickian diffusion, α obtained in the previous experiments for the open-face specimens was employed. The fitting results revealed a boundary condition of 100%. Table 10 presents the obtained diffusion coefficients compared with those in the bulk.

**Table 10:** Diffusion Coefficients and Delay Time of the Bulk and Adhesive Layer in Adhesives II and III Determined by Sequential Dual Fickian Diffusion

		Open-face			Closed (average)		
		Diffusion coefficient		Delay time	Diffusion coefficient		Delay time
Adhesive	Environment	*D*_1_ [mm^2^/h]	*D*_2_ [mm^2^/h]	*t*_d_ [h]	*D*_1_ [mm^2^/h]	*D*_2_ [mm^2^/h]	*t*_d_ [h]
II	100% RH	3.00 × 10^–4^	1.24 × 10^–5^	219	4.01 × 10^–4^	1.96 × 10^–5^	168
	Water immersion	3.05 × 10^–4^	1.71 × 10^–5^	149	4.24 × 10^–4^	2.69 × 10^–5^	82.8
III	100% RH	7.03 × 10^–4^	9.78 × 10^–6^	87.8	1.50 × 10^–3^	3.37 × 10^–5^	63.9
	Water immersion	1.03 × 10^–3^	1.89 × 10^–5^	50.9	1.16 × 10^–3^	5.18 × 10^–5^	9.23

**Figure 19:**
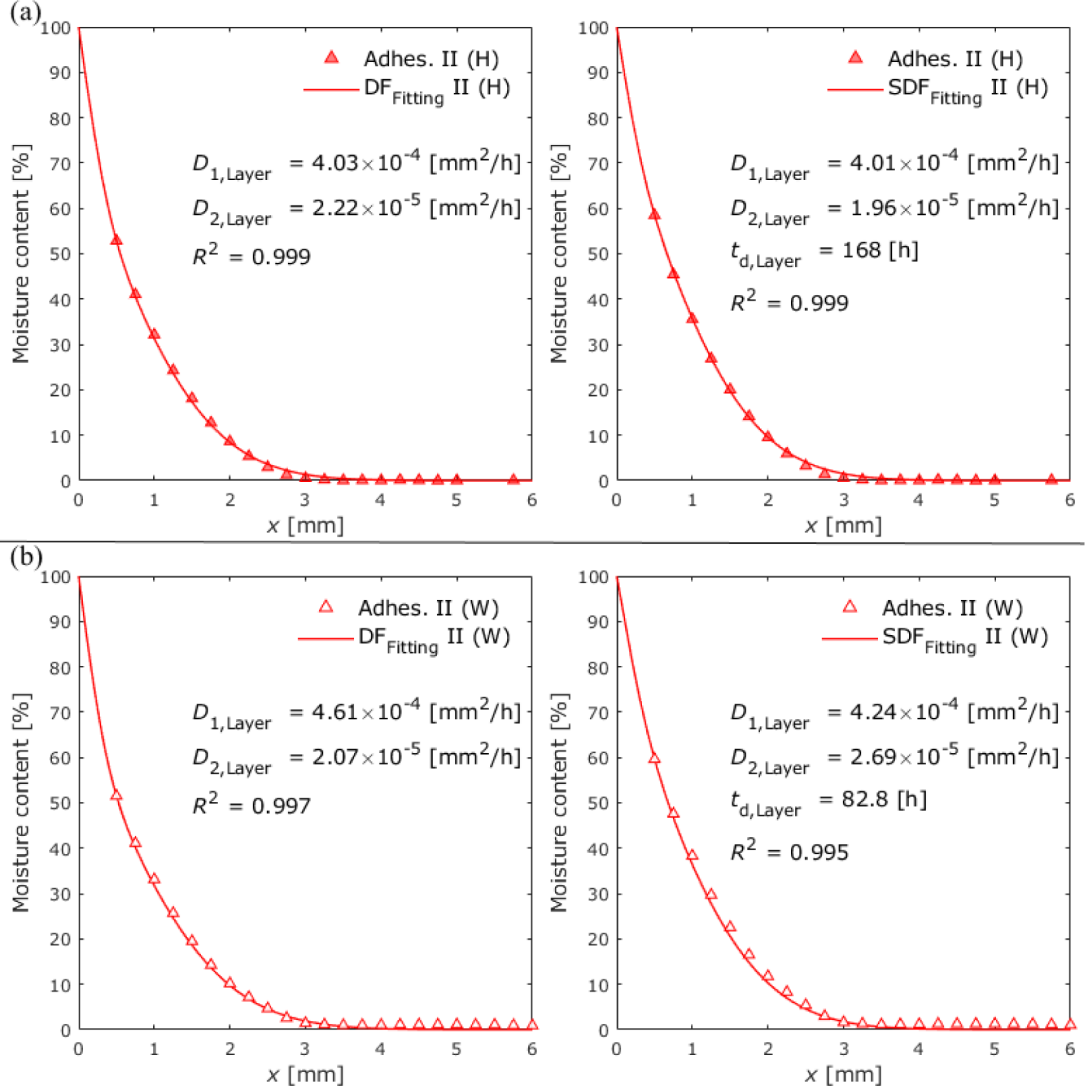
Average moisture distributions in closed specimens with Adhesive II at an exposure time of 12 weeks under (a) 100% RH exposure and (b) water immersion conditions.

**Figure 20:**
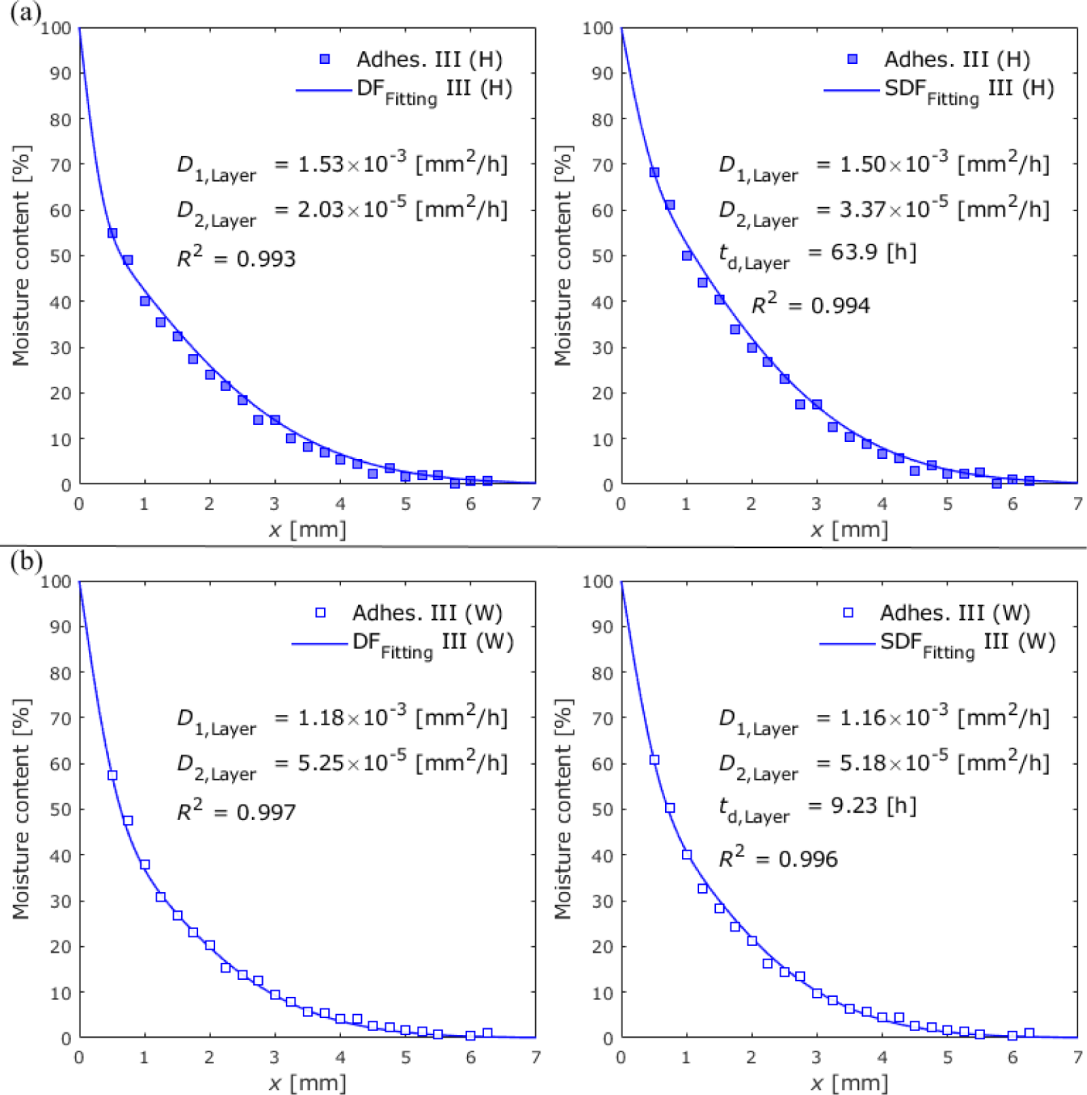
Average moisture distributions in closed specimens with Adhesive III at an exposure time of 12 weeks under (a) 100% RH exposure and (b) water immersion conditions.

Figures 19 and 20 show the moisture distributions determined by data fitting after 12 weeks of exposure for Adhesives II and III with dual Fickian and sequential dual Fickian fitting, respectively.

### 3.4 Diffusion Coefficient Difference in the Bulk and Adhesive Layer

**Figure 21:**
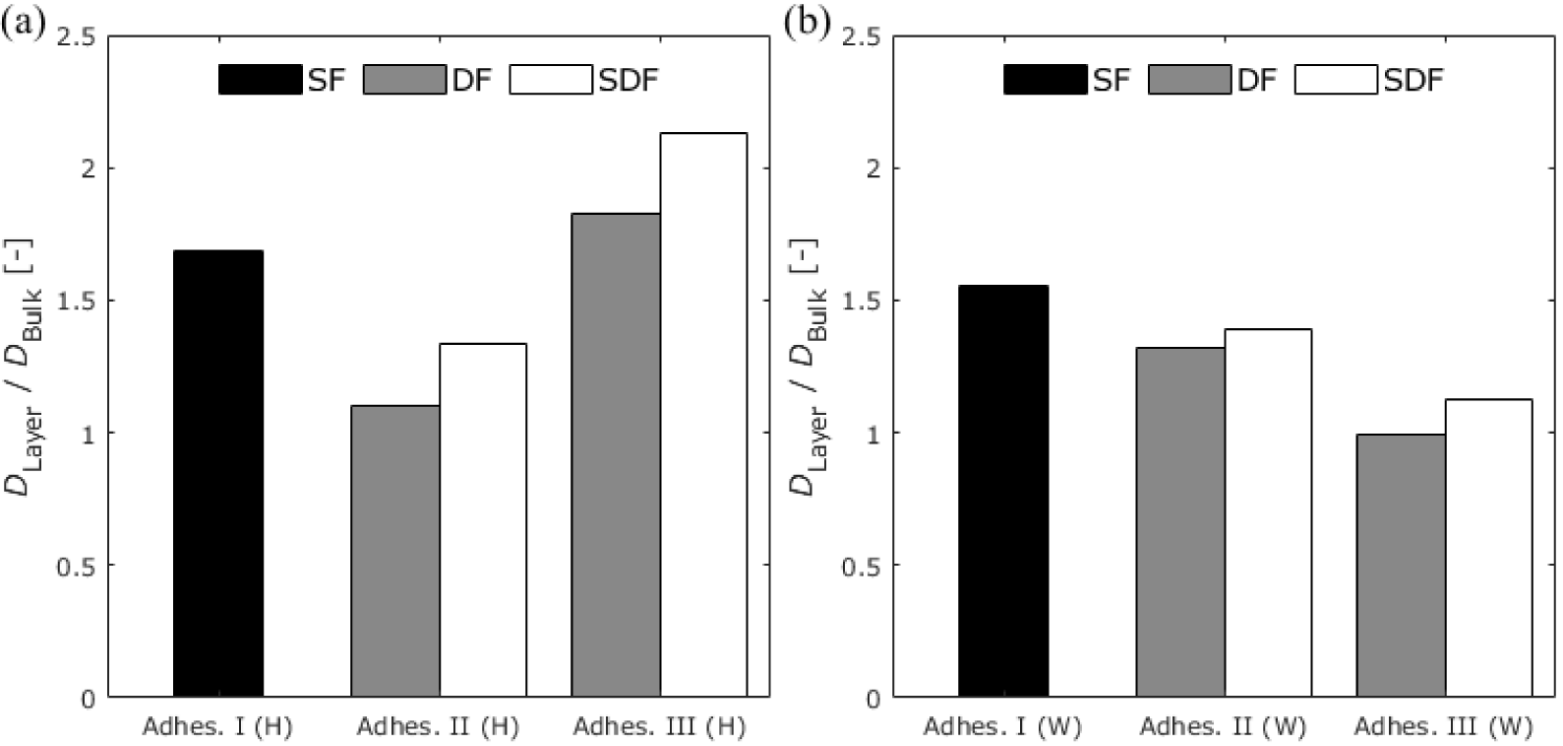
Change in the diffusion coefficient of first-stage diffusion between the bulk and adhesive layer under (a) 100% RH exposure and (b) water immersion conditions.

Figure 21 shows the change in the diffusion coefficient of first-stage diffusion between the bulk and adhesive layers. In most cases, the diffusion coefficient calculated from the moisture distribution in the adhesive layer was greater than that in the bulk, as calculated using the traditional gravimetric method. For Adhesive I, the results under both high-humidity exposure and water immersion conditions showed an approximately 1.5 times increase in the diffusion coefficient. In the case of Adhesive III, a different trend was observed under different exposure conditions. Under the high-humidity exposure conditions, the diffusion coefficient was approximately doubled, whereas it was almost the same under the water immersion condition. The former increase was believed to be because of the effect of the additives. Conversely, the latter change was attributed to differences in the ease of dissolution depending on the specimen type. For the open-face specimens, the diffusion coefficient differed between high-humidity exposure and water immersion, which was attributed to leaching. However, the migration of components in the adhesive was limited in the closed specimen. For closed specimens, the contact area between the adhesive and water was limited, thereby reducing leaching. Indeed, no significant differences in moisture distribution were observed between the high-humidity exposure and water immersion conditions, as shown in Figures 16, 19, and 20. The penetrated tip was approximately 4, 3, and 5 mm for Adhesives I, II, and III, respectively, which is consistent with the magnitude of the diffusion coefficient. Although Adhesive III had the lowest total moisture content in the bulk, it did not significantly affect the penetration depth.

There have been several discussions on why moisture penetrates the adhesive layer faster than that in the bulk. Zanni-Deffarges et al.^19^ proposed the concept of capillary diffusion and demonstrated that moisture diffusion near the interface between the adhesive and the substrate is faster than that at the center of the adhesive layer. Rapid moisture diffusion can be associated with the distribution of the curing agent in the thickness direction^50^ or the substrate wettability.^19,51^ These factors change the cross-link density near the interface, influencing the moisture diffusion at the interface/interphase^38^ Therefore, when an adhesive layer sandwiched between the substrates is exposed to a humid/water environment, rapid moisture diffusion near the interface can accelerate the overall moisture diffusion in the adhesive layer.^51^ However, in this study, moisture distribution through the thickness of the adhesive layer was not observed, and only the average moisture content through the thickness was detected. In addition, owing to the limited ability to detect small absorbances, the moisture that penetrated the interface between the adhesive and substrate may have been overlooked. Therefore, further investigations are required to clarify the interface moisture penetration.

## 4 Conclusions

In this study, we investigated the effects of curing agents and additives on moisture diffusion in adhesive layers using fiber-type NIRS. The adhesive layers were exposed to 100% RH and immersed in water to investigate the effect of environmental exposure. First, the diffusion coefficients were determined from the gravimetric changes of the epoxy-coated quartz glass plate specimens. We found that the diffusion behavior varied according to the curing agent used. Furthermore, when additives were included in the adhesive, it exhibited a greater diffusion coefficient than that composed only of the resin and curing agent, despite exhibiting a lower moisture content. Next, we measured the moisture distribution in the adhesive layers sandwiched between two quartz glass plates. To determine the diffusion coefficient in the adhesive layer, the classical distribution equation was modified, and the modified equations were fitted to the measured moisture distribution. The diffusion coefficient of the adhesive layer was greater than that of the bulk, except when the adhesive with additives was immersed in water. In summary, the moisture diffusion behavior of epoxy adhesives can vary significantly depending on the characteristics of the curing agent and additives. Furthermore, the moisture diffusion in the adhesive layer tends to be faster than that in the bulk. Therefore, it is crucial to consider not only diffusion in the bulk but also rapid diffusion at the interfaces/interphases. Further research is needed to elucidate moisture penetration near the interface for a more accurate analysis of moisture diffusion in the adhesive layer.

## Data Availability

Data supporting the findings of this study are available from the corresponding author upon request.

## Supporting Information Available

S1 Epoxy adhesives with amine-based curing agents: chemical structure and reaction of adhesives. S2 Curing condition of Adhesive I. S3 Curing conditions of Adhesives II and III (PDF)

## Abbreviations

NIRS: near-infrared spectroscopy
CTBN: carboxyl-terminated butadiene acrylonitrile
DICY: Dicyandiamide
RH: relative humidity
SF: simple Fickian
DF: dual Fickian
SDF: sequential dual Fickian
