# Supplementary material for: Direct Measurement of the Diffusion Coefficient of Adhesives from Moisture Distribution in Adhesive Layers Using Near-Infrared Spectroscopy
Source: ACS Appl Mater Interfaces. 2024 Sep 28;16(40):54611–27. doi: 10.1021/acsami.4c11286 (PMC11472270; doi:10.1021/acsami.4c11286)
Supplement: Supplementary file 1 — am4c11286_si_001.pdf [file am4c11286_si_001.pdf]

Supporting Information

Direct Measurement of the Diffusion Coefficient of Adhesives from Moisture Distribution in Adhesive Layers Using Near-Infrared Spectroscopy

Jin-Woo Han[1,*], *Yu Sekiguchi*[2,*], *Kazumasa Shimamoto*[3], *Haruhisa Akiyama*[3], *Chiaki Sato*[2]

[1] Department of Mechanical Engineering, Tokyo Institute of Technology, 4259 Nagatsuta-cho, Midori-ku, Yokohama 226-8501, Kanagawa, Japan

[2] Institute of Innovative Research, Tokyo Institute of Technology, 4259 Nagatsuta-cho, Midori-ku, Yokohama 226-8501, Kanagawa, Japan

[3] Nanomaterials Research Institute, National Institute of Advanced Industrial Science and Technology (AIST), 1-1-1 Higashi, Tsukuba 305-8565, Ibaraki, Japan

Email

J.-W. Han: han.j.aj@m.titech.ac.jp; Y. Sekiguchi: sekiguchi.y.aa@m.titech.ac.jp

S1 Epoxy adhesives with amine-based curing agents

In all the specimens, bisphenol A epoxy resin was used, and the chemical structure is shown in Figure S1(a). The chemical structures of poly(propylene glycol) bis(2-aminopropyl ether) and dicyandiamide (DICY), which are used in this study as curing agent, are shown in Figures S1(b) and S1(c).

The basic reaction of bisphenol A epoxy resin with amine-based curing agents is shown in Figure S2. When an amine-based curing agent is used with bisphenol A epoxy resin, the nitrogen of the amine initiates a nucleophilic attack on the carbon of the epoxy ring.[52] This reaction results in the opening of the epoxy ring, forming a hydroxyl group while creating a new C-N bond. Each amine molecule possesses more than one reactive site, allowing a single amine molecule to react with multiple epoxy molecules simultaneously, leading to cross-linking. This cross-linking process forms a three-dimensional network, gradually hardening the polymer. Since the reaction with amine curing agents is exothermic, the curing rate can be controlled by the external temperature and the properties of the curing agent.

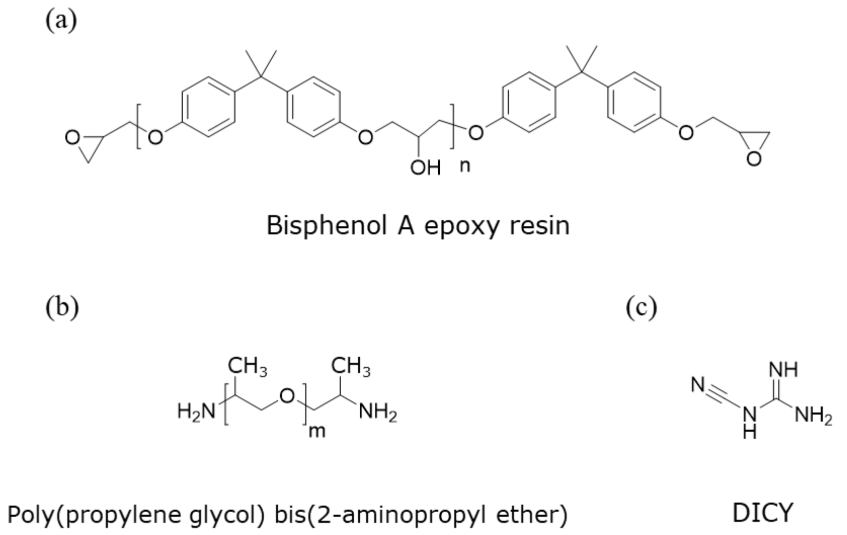

Figure S1. Chemical structure of (a) bisphenol A epoxy resin, (b) poly(propylene glycol) bis(2-aminopropyl ether), and (c) dicyandiamide.

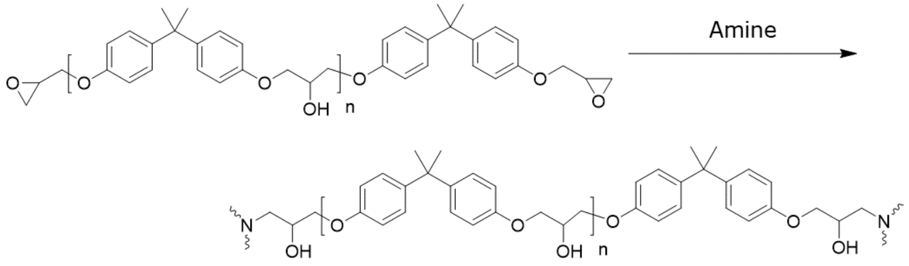

Figure S2. The reaction of bisphenol A epoxy resin with amine-based curing agents.

S2 Curing condition of adhesive I

The epoxy equivalent weight of bisphenol A epoxy resin (jER 828) is from 184 to 194 g/eq. The amine hydrogen equivalent weight (AHEW) of poly(propylene glycol) bis(2-

aminopropyl ether) is approximately 60 g/eq. The ratio of resin to curing agent was determined to give equal equivalent weights.

Poly(propylene glycol) bis(2-aminopropyl ether) allows the resin to be cured with heat or at room temperature. Heat curing causes some discoloration, but room-temperature curing produces a nearly transparent material. For room-temperature curing, the curing time is often set to approximately a week.[53,54] Differential scanning calorimetry (DSC) results are shown in Figure S3. Samples subjected to different curing times were placed in a DSC-60 Plus (Shimadzu Corp., Kyoto Japan) and tested at a heating rate of 10 °C /min. The peak decreased with increasing curing time, with approximately 85 % reduction in the peak area after a week.

S3 Curing conditions of adhesives II and III

Adhesive II was prepared for comparison with Adhesive III, whose composition was known as disclosed by the manufacturer. Therefore, the resin to curing agent ratio of adhesive II was determined to be the same ratio as that of adhesive III.

The curing condition of adhesive III was set to 180 °C for 1 h as recommended by the manufacturer. However, carbonization occurred under the same curing condition for adhesive II. It is believed that the adhesive did not contain any additives, which caused it to over-cure, resulting in thermal runaway. In the case of a latent curing agent such as DICY, the curing reaction occurs instantaneously from reaching initiation conditions. Therefore, the curing time was reduced to 30 min for adhesive II to obtain a nearly transparent material.

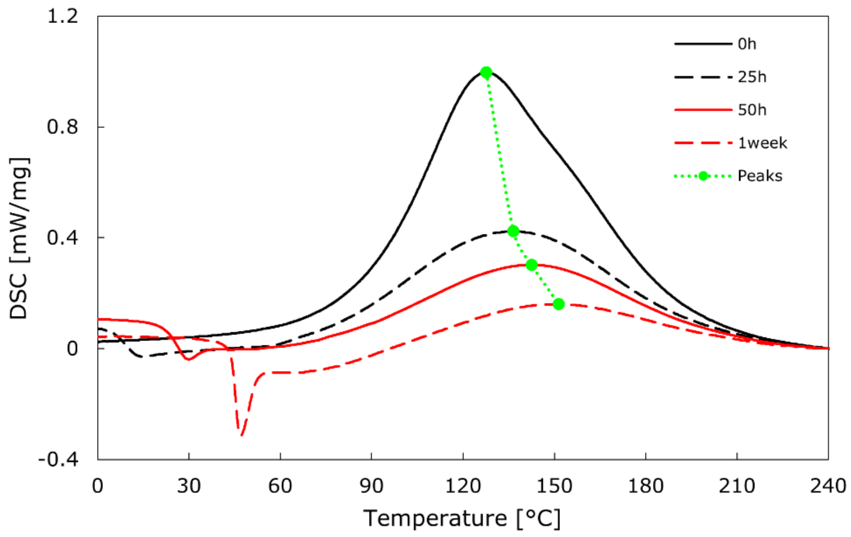

Figure S3. Non-isothermal DSC heating curves of Adhesive I according to the curing time. The heating rate was set to 10 °C/min.

REFERENCES

52. Bashir, M.A. Cure Kinetics of Commercial Epoxy-Amine Products with Iso-Conversional Methods. *Coatings* **2023**, 13, 592. DOI: 10.3390/coatings13030592.

53. Yoon, I.-N.; Lee, Y.; Kang, D.; Min, J.; Won, J.; Kim, M.; Kang, Y.S.; Kim, S.-H.; Kim, J.J. Modification of hydrogenated Bisphenol A epoxy adhesives using nanomaterials. *Int. J. Adhes. Adhes.* **2011**, 31, 119-125. DOI: 10.1016/j.ijadhadh.2010.11.010.

54. Burton, B.; Alexander, D.; Klein, H.; Garibay-Vasquez, A.; Pekarik A.; Henkee, C. *Epoxy formulations using Jeffamine® polyetheramines*, Huntsman Corp., The Woodlands, TX, 2005.
